# Characterization of the Epidemiological Profile of Patients With Parkinson's Disease Who Were Hospitalized due to SARS‐CoV‐2 Infection: A Portrait of 4 Years of the Pandemic in Brazil

**DOI:** 10.1002/jmv.70670

**Published:** 2025-11-03

**Authors:** Adriele Evelyn Ferreira Silva, Patrícia Teixeira Costa, Lucas Silva Mello, Luiz Felipe Azevedo Marques, Fernando Augusto Lima Marson

**Affiliations:** ^1^ Laboratory of Molecular Biology and Genetics, Postgraduate Program of Health Sciences, Postgraduate Program in Health Data Science São Francisco University Bragança Paulista São Paulo Brazil; ^2^ Laboratory of Clinical and Molecular Microbiology, Postgraduate Program of Health Sciences, Postgraduate Program in Health Data Science São Francisco University Bragança Paulista São Paulo Brazil; ^3^ LunGuardian Research Group—Epidemiology of Respiratory and Infectious Diseases, Postgraduate Program of Health Sciences, Postgraduate Program in Health Data Science São Francisco University Bragança Paulista São Paulo Brazil

**Keywords:** COVID‐19, epidemiology, neurodegeneration, Parkinson's disease, public health

## Abstract

Individuals with Parkinson's disease may be more susceptible to severe coronavirus disease (COVID)‐19 outcomes due to advanced age and neurological and systemic impairments. However, the evidence on this association remains inconclusive. This study investigates the impact of severe acute respiratory syndrome coronavirus 2 infection in hospitalized patients with Parkinson's disease. Secondary data from the Influenza Epidemiological Surveillance Information System were obtained from the OpenData‐SUS platform of the Brazilian Ministry of Health, covering March 2020 to March 2024. Variables included demographics, comorbidities, clinical signs and symptoms, ventilatory and intensive care unit (ICU) support, hospital stay, and outcomes. Missing data were imputed, and descriptive, bivariate, and multivariable analyses were conducted using 5% significance level. Among 1 725 690 hospitalized COVID‐19 patients in Brazil, 4907 (0.3%) had Parkinson's disease. Overall mortality was 37.2%, while mortality among Parkinson's patients reached 53.1% (OR = 1.920; 95% CI = 1.815–2.031), indicating a significantly higher risk. Death was independently associated with advanced age, male sex, low vaccination coverage, and cardiovascular or chronic respiratory diseases. Age strongly predicted both Parkinson's diagnosis and mortality. Parkinson's patients had higher ICU admission rates and more frequent need for invasive ventilation. Multivariable analysis confirmed Parkinson's disease as an independent mortality risk factor (OR = 1.277; 95% CI = 1.198–1.360). The logistic regression model for mortality demonstrated good discrimination, with an area under the receiver operating characteristic curve of 0.810 (95% CI = 0.809–0.811), indicating reliable performance in distinguishing patients who experienced the outcome. Parkinson's disease is associated with higher mortality and greater ICU needs in hospitalized COVID‐19 patients. These findings underscore the need for targeted prevention, close monitoring, and tailored clinical management for this vulnerable group, especially considering their age and comorbid conditions.

## 1 Introduction

The coronavirus disease (COVID)‐19 pandemic has affected millions of people around the world, resulting in more than seven million deaths [1]. The challenges of dealing with the severe acute respiratory syndrome coronavirus 2 (SARS‐CoV‐2) infection have had a disproportionate impact on healthcare systems and on individuals with pre‐existing comorbidities such as neurodegenerative diseases [2]. Advanced age is the main risk factor for adverse clinical outcomes associated with COVID‐19 due to changes in the immune system and respiratory system function resulting from systemic aging [3]. Parkinson's disease is the second most common neurodegenerative disease in the elderly population, behind only Alzheimer's disease, with more than six million cases registered worldwide [4]. The incidence of Parkinson's disease increases 5–10 times between the sixth and ninth decades of life, and its prevalence also increases with advancing age [5].

Parkinson's disease is a progressive neurodegenerative disease characterized by the loss of dopaminergic neurons in the substantia nigra, located in the midbrain, and by the presence of insoluble aggregates of alpha‐synuclein, known as Lewy bodies [5]. This condition is marked by motor alterations, including bradykinesia (decreased speed of movement), rigidity (increased muscle tone), tremor, and alterations in gait and postural reflexes [4]. In addition to motor symptoms, Parkinson's disease also involves non‐motor symptoms such as speech impairment and dysphagia, olfactory dysfunction, psychiatric symptoms, cognitive impairment, and autonomic dysfunctions such as chronic constipation, incontinence, and symptomatic orthostatic hypotension whose symptoms tend to worsen with the progression of the disease [6]. Gastrointestinal dysfunction is recognized as one of the most prominent non‐motor symptoms of Parkinson's disease, with constipation often preceding motor manifestations by decades. Evidence indicates that intestinal microbiota dysbiosis represents a key determinant in the pathogenesis of Parkinson's disease [7, 8, 9]. Alterations in the gut microbiome and related metabolites may modulate pathophysiological mechanisms involved in disease progression. In this context, the literature has demonstrated that probiotics exert antioxidant effects, improving mitochondrial dynamics and maintaining cellular homeostasis, thereby contributing to the attenuation of clinical impacts associated with Parkinson's disease [10].

Although SARS‐CoV‐2 mainly binds to the epithelial cells of the respiratory tract, the rapid replication of the virus in the lungs can trigger an intense immune response [11]. This deregulated host inflammatory response leads to the cytokine storm and aggressive inflammation, which can cause collateral tissue damage and culminate in systemic failure. The most common symptoms of infection include fever, dry cough, fatigue, myalgia, and dyspnea [11]. SARS‐CoV‐2 infection also induces significant alterations in the gut microbiota, contributing to systemic inflammation, immune dysregulation, and worse clinical outcomes. Dysbiosis plays a central role in COVID‐19 progression and long‐COVID, although the underlying mechanisms remain unclear [12]. In addition, neurological manifestations have also been described, ranging from mild headache, nausea, vomiting, and even generalized convulsions and altered consciousness. Less frequent manifestations include seizures, strokes of multiple etiologies, and Guillain‐Barré syndrome [13, 14]. These neurological manifestations can exacerbate previous symptoms of chronic neurological conditions due to indirect damage caused by SARS‐CoV‐2 infection.

Through bioinformatics analyses, Shi et al. [15] identified 52 commonly differentially expressed genes among Alzheimer's disease, Parkinson's disease, and COVID‐19, with 5 hub genes highlighted in the protein–protein interaction network: *GAD2* (Glutamate Decarboxylase 2), *SST* (Somatostatin), *TAGLN3* (Transgelin 3), *SYP* (Synaptophysin), and *KCNJ4* (Potassium Inwardly Rectifying Channel Subfamily J Member 4). These analyses indicated that the synaptic vesicle cycle represents a shared molecular pathway across these conditions [15]. Subsequent evaluations suggested that infection with SARS‐CoV‐2 may induce synaptic dysfunction, characterized by widespread downregulation of synaptic proteins in the cerebral cortex, potentially triggering or accelerating neurodegenerative processes [15]. Additional studies further support that protein–protein interactions between SARS‐CoV‐2 and host proteins may contribute to the onset or progression of Parkinson's disease [16, 17].

Nevertheless, the literature still presents conflicting results regarding the relationship between Parkinson's disease and COVID‐19. A study suggests that the inflammatory mechanisms involved in both conditions can lead to neurodegeneration, and it is hypothesized that SARS‐CoV‐2 infection can accelerate the progression of Parkinson's disease, while the latter makes individuals more vulnerable to negative outcomes related to viral infection [18]. A systematic review showed that individuals with Parkinson's disease were more susceptible to SARS‐CoV‐2, with an increased risk of mortality associated with COVID‐19 [19]. A meta‐analysis characterized the COVID‐19 phenotype in patients with Parkinson's disease and described that this population was associated with the presence of obesity, lung disease, hospitalization, and death [20]. However, another systematic review that analyzed the prevalence and prognosis of COVID‐19 found no significant differences between the hospitalization rate and mortality of patients with Parkinson's disease and individuals without the disease [13].

Considering the divergent data in the literature and the importance of identifying the groups most vulnerable to SARS‐CoV‐2 infection and its adverse outcomes, this study aims to analyze the epidemiological profile of individuals with Parkinson's disease who were hospitalized due to SARS‐CoV‐2 infection, using a representative cohort of 4 years of follow‐up of the COVID‐19 pandemic in Brazil.

## 2 Methods

The epidemiological analysis was performed based on data made available by the Open‐Data‐SUS platform (https://opendatasus.saude.gov.br/), created by the Brazilian Ministry of Health to compile surveillance information on severe acute respiratory syndrome obtained by the Influenza Epidemiological Surveillance Information System (*Sistema de Informação da Vigilância Epidemiológica da Gripe*—SIVEP‐*Gripe*). The file was viewed using the Statistical Package for the Social Sciences (SPSS) software (IBM SPSS Statistics for Macintosh, Version 28.0). Data were extracted for the first 4 years of the COVID‐19 pandemic, covering the period from March 2020 to March 2024, according to the following information:

Demographic profile, including sex (male and female), race (White people, Black people, Asian individuals, Mixed individuals, and Indigenous peoples), place of residence (urban or rural + peri‐urban), and age groups (40–59 years, 60–64 years, 65–69 years, 70–74 years, 75–79 years, 80–84 years, 85–90 years, +90 years), considering the incidence of Parkinson's disease, which, although rare, can manifest as early as age 40 and whose prevalence increases with age [21].
Data on viral infection, such as residence in areas with an influenza outbreak, presence of hospital‐acquired (nosocomial) infection, and use of antivirals to treat influenza virus infection;
Presence of comorbidities, including any comorbidity (at least one), such as heart disease (cardiopathy), hematological disorders, Down syndrome, hepatic disorders, asthma, diabetes mellitus, chronic respiratory diseases, immunosuppressive disorders, kidney diseases, and obesity;
Clinical signs and symptoms related to the diagnosis of severe acute respiratory syndrome, such as fever, cough, sore throat, dyspnea, respiratory discomfort, oxygen saturation < 95%, diarrhea, vomiting, fatigue, and other symptoms;
COVID‐19 vaccination status;
Need for intensive care unit treatment and need for mechanical ventilation support (invasive mechanical ventilation, noninvasive mechanical ventilation, and no need for mechanical ventilation);
Length of hospital stay (in days);
Length of stay in the intensive care unit (days); and
Outcomes, including hospital discharge or deaths.

To ensure the accuracy of the data, the categorical variables were coded numerically, enabling the analysis of missing data, as well as descriptive and inferential statistical analysis.

The data set was exported from SPSS to an .xls file to impute the missing data using XLSTAT Statistical Software for Excel. Patient characteristics with more than 40% missing data were excluded due to methodological and practical considerations. Most of these variables did not follow a *Missing Completely at Random* pattern, limiting the reliability of imputation. Although our sample size allowed reasonably accurate imputation, including variables with > 40% missingness could increase bias rather than improve robustness. Variables with moderate missingness ( < 40%) were retained because they were strongly correlated with other clinical and demographic variables, allowing reliable estimation of missing values through imputation.

The following methods were used to impute the data: qualitative (categorical) data were estimated using the NIPALS (Nonlinear Iterative Partial Least Squares) algorithm, while quantitative (numerical) data were imputed using the MCMC (Markov Chain Monte Carlo) multiple imputation algorithm. After imputation, XLSTAT Statistical Software generated a new data set in .xls format, which was used to conduct the statistical analyses in SPSS software, including descriptive and inferential analyses.

Bivariate statistical analysis was conducted using SPSS software. We used the Logistic Regression test to evaluate the frequency of deaths and the presence of Parkinson's disease in relation to the epidemiological data of individuals infected with SARS‐CoV‐2. To estimate the association of each marker with the outcome of death and with the diagnosis of Parkinson's disease, the odds ratio (OR) and the 95% confidence interval (95% CI) were calculated. Statistical analysis to associate the evaluated time intervals with the likelihood of classifying the patient as having Parkinson's and with the likelihood of death was performed using the Student's *t*‐test.

The multivariable analysis was conducted using the Binary Logistic Regression Model with the Stepwise Backward method. As an initial inclusion criterion in the model, markers with *p* ≤ 0.05 were selected in the bivariate analysis. The dependent variable (response) in the model was the outcome (hospital discharge or death) or the presence of Parkinson's disease. In the Logistic Regression Model, we present: (i) the B coefficient (including the standard error [SE], and for the constant it is called the intercept); (ii) the Wald chi‐square test and its significance; (iii) the degrees of freedom for the Wald chi‐square test; and (iv) Exp(B), which represents the exponentiation of the B coefficient (OR), including its 95% CI. Before conducting the analysis, we tested the markers for multicollinearity, adopting the following criteria: tolerance < 0.1 and variance inflation factor > 10. The discrimination ability of each model was assessed by calculating the area under the receiver operating characteristic curve (AUC).

Diagnostic performance metrics were calculated by comparing patients’ clinical and demographic characteristics with the presence of a Parkinson's disease diagnosis and clinical outcomes. Analyses were conducted using the MedCalc Diagnostic Test Evaluation Calculator (MedCalc Software Ltd., Version 23.3.4). The software computed sensitivity, specificity, predictive values, and overall accuracy, along with corresponding 95% CIs. Exact CIs for sensitivity, specificity, and accuracy were estimated using the Clopper‐Pearson method. Predictive values were reported with standard logit intervals, except when results equaled 0% or 100%, in which case exact Clopper‐Pearson intervals were applied. A two‐sided significance level of 0.05 was adopted for all statistical analyses.

The figures were generated in GraphPad Prism, version 10.2.3 for Mac (GraphPad Software, San Diego, California, USA, http://www.graphpad.com).

Data collection was restricted to the defined study period to ensure reliability and completeness of the analyses. Although more recent data are publicly available, they are subject to substantial limitations due to the significant reduction in COVID‐19 testing in recent years, including among hospitalized patients with clinical features of infection. Additionally, despite the public accessibility of the database, extensive manual corrections and adjustments are necessary before definitive analysis, which contributes to the interval between data acquisition and publication.

## 3 Results

### 3.1 Hospitalizations due to SARS‐CoV‐2 in Brazil: Geographical Distribution and Demographic Characterization of Patients

The study included 1 725 690 individuals aged 40 and over hospitalized due to SARS‐CoV‐2 infection in Brazil. The distribution of patients according to the federative unit of residence, notification, and hospitalization is detailed in Table S1. Regarding the place of notification, there was a higher prevalence of cases in the Southeast [*N* = 879 296 (51.0%)], followed by the South [*N* = 300 061 (17.4%)], Northeast [*N* = 268 306 (15.6%)], Central West [*N* = 171 994 (9.9%)], and North [*N* = 106 033 (6.2%)]. The distribution of patients according to the federative unit of residence and hospitalization showed a similar pattern to that observed for the federative unit of notification.

When analyzing the population, there was a predominance of male patients [*N* = 945 745 (54.8%)], people who declared themselves White [*N* = 1 033 674 (59.9%)], and living in urban areas [*N* = 1 644 128 (95.3%)], as shown in Table 1. As for age, the largest portion of the population is between 40 and 59 years old [*N* = 693 637 (40.2%)], followed by 60–64 years old [*N* = 197 781 (11.5%)], 65–69 years old [*N* = 198 798 (11.5%)], 70–74 years [*N* = 183 947 (10.7%)], 75–79 years [*N* = 156 156 (9.0%)], 80–84 years [*N* = 131 393 (7.6%)], 85–90 years [*N* = 94 082 (5.5%)], and over 90 years [*N* = 69 896 (4.1%)] (Table 1).

**Table 1:** Demographic characteristics, clinical signs and symptoms, comorbidities, and hospitalization data of individuals hospitalized due to severe acute respiratory syndrome coronavirus 2 (SARS‐CoV‐2) infection in Brazil.

Markers	Categories	*N* (%)
Parkinson's disease	Yes	4907 (0.3%)
	No	1 720 783 (99.7%)
Vaccination against coronavirus disease (COVID)‐19	Yes	401 940 (23.3%)
	No	1 323 750 (76.7%)
Sociodemographic		
Sex	Male	945 745 (54.8%)
	Female	779 945 (45.2%)
Age	40–59 years of age	693 637 (40.2%)
	60–64 years of age	197 781 (11.5%)
	65–69 years of age	198 798 (11.5%)
	70–74 years of age	183 947 (10.7%)
	75–79 years of age	156 156 (9.0%)
	80–84 years of age	131 393 (7.6%)
	85–90 years of age	94 082 (5.5%)
	+90 years of age	69 896 (4.1%)
Race	White people	1 033 674 (59.9%)
	Black people	74 171 (4.3%)
	Asian individuals	17 254 (1.0%)
	Mixed individuals^a^	597 696 (34.6%)
	Indigenous peoples	2895 (0.2%)
Geographic zone	Urban	1 644 128 (95.3%)
	Rural + peri‐urban	81 562 (4.7%)
Nosocomial infection	Yes	35 122 (2.0%)
	No	1 690 568 (98.0%)
Clinical signs and symptoms	Fever	1 233 742 (71.2%)
	Cough	1 406 301 (81.5%)
	Sore throat	285 314 (16.5%)
	Dyspnea	1 435 753 (83.2%)
	Respiratory discomfort	1 303 532 (75.5%)
	Oxygen saturation < 95%	1 387 616 (80.4%)
	Diarrhea	211 357 (12.2%)
	Vomiting	128 997 (7.5%)
	Fatigue	383 315 (22.2%)
	Other symptoms	552 153 (32.0%)
Comorbidities	Heart disease (cardiopathy)	629 158 (36.5%)
	Hematological disorder	11 715 (0.7%)
	Down syndrome	3507 (0.2%)
	Hepatic disorder	14 565 (0.8%)
	Asthma	38 417 (2.2%)
	Diabetes mellitus	443 509 (25.7%)
	Chronic respiratory disease	66 654 (3.9%)
	Immunosuppression disorder	41 510 (2.4%)
	Kidney disease	66 795 (3.9%)
	Obesity	128 186 (7.4%)
	Other comorbidities	498 787 (28.9%)
Received antiviral medication for the flu	Yes	111 583 (6.5%)
	No	1 614 107 (93.5%)
Need for intensive care unit	Yes	591 959 (34.3%)
	No	1 133 731 (65.7%)
Need to mechanical ventilatory support	Invasive	326 963 (18.9%)
	Noninvasive	1 107 497 (64.2%)
	No	291 230 (16.9%)
Outcome	Hospital discharge	1 084 333 (62.8%)
	Death	641 357 (37.2%)

*Note:* Data are presented as absolute frequencies (*N*) and relative frequencies (%, percentages). The epidemiological analysis was based on data from Open‐Data‐SUS (https://opendatasus.saude.gov.br/), covering a 4‐year period of the COVID‐19 pandemic in Brazil (from February 22, 2020 to May 24, 2024).

^a^ Individuals with a multiracial background (*Pardos*).

Respiratory signs and symptoms were the most prevalent among those recorded, especially dyspnea [*N* = 1 435 753 (83.2%)], cough [*N* = 1 406 301 (81.5%)], peripheral oxygen saturation < 95% [*N* = 1 387 616 (80.4%)], and respiratory discomfort [*N* = 1 303 532 (75.5%)], as shown in Table 1. The most frequent comorbidities were heart disease (cardiopathy) [*N* = 629 158 (36.5%)] and diabetes mellitus [*N* = 443 509 (25.7%)] (Table 1). The presence of Parkinson's disease was identified in 4907 hospitalized patients, corresponding to 0.3% of the sample (Table 1).

In total, 111 583 individuals (6.5%) used antiviral drugs to manage flu symptoms (Table 1). Hospitalization in an intensive care unit was necessary in 591 959 cases (34.3%) (Table 1). The majority of hospitalized patients required ventilatory support, with 326 963 (18.9%) undergoing invasive ventilation and 1 107 497 (64.2%) noninvasive ventilation. Death was observed in 641 357 patients (37.2%) (Table 1).

### 3.2 Geographical Distribution and Factors Associated With the Diagnosis of Parkinson's Disease in Patients Hospitalized for SARS‐CoV‐2 Infection

When comparing the distribution of patients between the country's macro‐regions, considering the place of notification and the diagnosis of Parkinson's disease, there is a modest difference between cases with Parkinson's and the general hospitalized population (Table 2). In general, the highest prevalence of patients occurred in the Southeast [*N* = 876 790 (51.0%)], followed by the South [*N* = 299 156 (17.4%)], Northeast [*N* = 267 517 (15.6%)], Central West [*N* = 171 457 (9.9%)], and North [*N* = 105 863 (6.2%)]. Following the same pattern, the highest concentration of patients diagnosed with Parkinson's was recorded in the Southeast [*N* = 2506 (51.1%)], followed by the South [*N* = 905 (18.4%)], Northeast [*N* = 789 (16.1%)], Central West [*N* = 537 (11.0%)], and North [*N* = 170 (3.4%)]. The distribution of patients based on the federative unit of residence and hospitalization was similar to that of notification.

**Table 2:** Places of residence, case notification, and hospitalization of individuals hospitalized due to severe acute respiratory syndrome coronavirus 2 (SARS‐CoV‐2) infection in Brazil according to Parkinson's disease diagnosis.

Federative units	Place of residence		Place of notification		Place of hospitalization	
	Parkinson	Control	Parkinson	Control	Parkinson	Control
Central‐West region	538 (11.0%)	171 431 (10.1%)	537 (11.0%)	171 457 (9.9%)	523 (10.7%)	169 250 (9.8%)
Federal District	140 (2.9%)	38 823 (2.3%)	142 (2.9%)	41 764 (2.4%)	135 (2.8%)	41 104 (2.4%)
Goiás	212 (4.3%)	71 514 (4.2%)	210 (4.3%)	69 357 (4.0%)	207 (4.2%)	68 569 (4.0%)
Mato Grosso	83 (1.7%)	30 801 (1.8%)	83 (1.7%)	30 354 (1.8%)	79 (1.6%)	29 813 (1.7%)
Mato Grosso do Sul	103 (2.1%)	30 293 (1.8%)	102 (2.1%)	29 982 (1.7%)	102 (2.1%)	29 794 (1.7%)
North region	173 (3.6%)	107 563 (6.3%)	170 (3.4%)	105 863 (6.2%)	162 (3.2%)	104 367 (6.1%)
Acre	7 (0.1%)	4290 (0.2%)	7 (0.1%)	4336 (0.3%)	7 (0.1%)	4272 (0.2%)
Amapá	6 (0.1%)	4402 (0.3%)	6 (0.1%)	4531 (0.3%)	6 (0.1%)	4500 (0.3%)
Amazonas	73 (1.5%)	32 131 (1.9%)	73 (1.5%)	31 347 (1.8%)	66 (1.3%)	31 003 (1.8%)
Pará	47 (1.0%)	41 744 (2.4%)	44 (0.9%)	40 996 (2.4%)	43 (0.9%)	40 469 (2.4%)
Rondônia	9 (0.2%)	12 984 (0.8%)	7 (0.1%)	12 685 (0.7%)	7 (0.1%)	12 518 (0.7%)
Roraima	4 (0.1%)	3588 (0.2%)	4 (0.1%)	3545 (0.2%)	4 (0.1%)	3540 (0.2%)
Tocantins	27 (0.6%)	8424 (0.5%)	29 (0.6%)	8423 (0.5%)	29 (0.6%)	8065 (0.5%)
Northeast region	790 (16.1%)	267 743 (15.6%)	789 (16.1%)	267 517 (15.6%)	766 (15.5%)	262 272 (15.2%)
Alagoas	55 (1.1%)	17 441 (1.0%)	53 (1.1%)	17 336 (1.0%)	52 (1.1%)	17 077 (1.0%)
Bahia	170 (3.5%)	62 275 (3.6%)	170 (3.5%)	62 102 (3.6%)	167 (3.4%)	60 909 (3.5%)
Ceará	192 (3.9%)	58 787 (3.4%)	194 (4.0%)	58 840 (3.4%)	184 (3.7%)	56 774 (3.3%)
Maranhão	27 (0.6%)	19 194 (1.1%)	26 (0.5%)	18 631 (1.1%)	25 (0.5%)	18.394 (1.1%)
Paraíba	108 (2.2%)	23 469 (1.4%)	106 (2.2%)	23 484 (1.4%)	105 (2.1%)	23 218 (1.3%)
Pernambuco	104 (2.1%)	42 459 (2.5%)	105 (2.1%)	42 424 (2.5%)	100 (2.0%)	41 591 (2.4%)
Piauí	59 (1.2%)	17 018 (1.0%)	59 (1.2%)	17 497 (1.0%)	59 (1.2%)	17 353 (1.0%)
Rio Grande do Norte	51 (1.0%)	16 570 (1.0%)	51 (1.0%)	16 570 (1.0%)	51 (1.0%)	16 462 (1.0%)
Sergipe	24 (0.5%)	10 530 (0.6%)	25 (0.5%)	10 633 (0.6%)	23 (0.5%)	10 494 (0.6%)
Southeast region	2501 (51.0%)	874 615 (50.9%)	2506 (51.1%)	876 790 (51.0%)	2410 (49.1%)	862 613 (50.1%)
Espírito Santo	24 (0.5%)	13 202 (0.8%)	23 (0.5%)	13 108 (0.8%)	22 (0.4%)	12 696 (0.7%)
Minas Gerais	594 (12.1%)	187 811 (10.9%)	598 (12.2%)	187 118 (10.9%)	577 (11.8%)	185 162 (10.8%)
Rio de Janeiro	398 (8.1%)	156 355 (9.1%)	398 (8.1%)	156 131 (9.1%)	382 (7.8%)	153 457 (8.9%)
São Paulo	1485 (30.3%)	517 247 (30.1%)	1487 (30.3%)	520 433 (30.2%)	1429 (29.1%)	511 298 (29.7%)
South region	905 (18.4%)	299 187 (17.4%)	905 (18.4%)	299 156 (17.4%)	889 (18.1%)	296 178 (17.2%)
Paraná	348 (7.1%)	117 187 (6.8%)	348 (7.1%)	117 191 (6.8%)	345 (7.0%)	116 569 (6.8%)
Rio Grande do Sul	301 (6.1%)	112 060 (6.5%)	300 (6.1%)	111 881 (6.5%)	293 (6.0%)	110 465 (6.4%)
Santa Catarina	256 (5.2%)	69 940 (4.1%)	257 (5.2%)	70 084 (4.1%)	251 (5.1%)	69 144 (4.0%)
Unknown (not informed)	0 (0.0%)	244 (0.0%)	None	None	157 (3.2%)	26 073 (1.5%)

*Note:* Data are presented as absolute frequencies (*N*) and relative frequencies (%, percentages). The epidemiological analysis was based on data from Open‐Data‐SUS (https://opendatasus.saude.gov.br/), covering a 4‐year period of the coronavirus disease (COVID)‐19 pandemic in Brazil (from February 22, 2020 to May 24, 2024).

**Figure 1:**
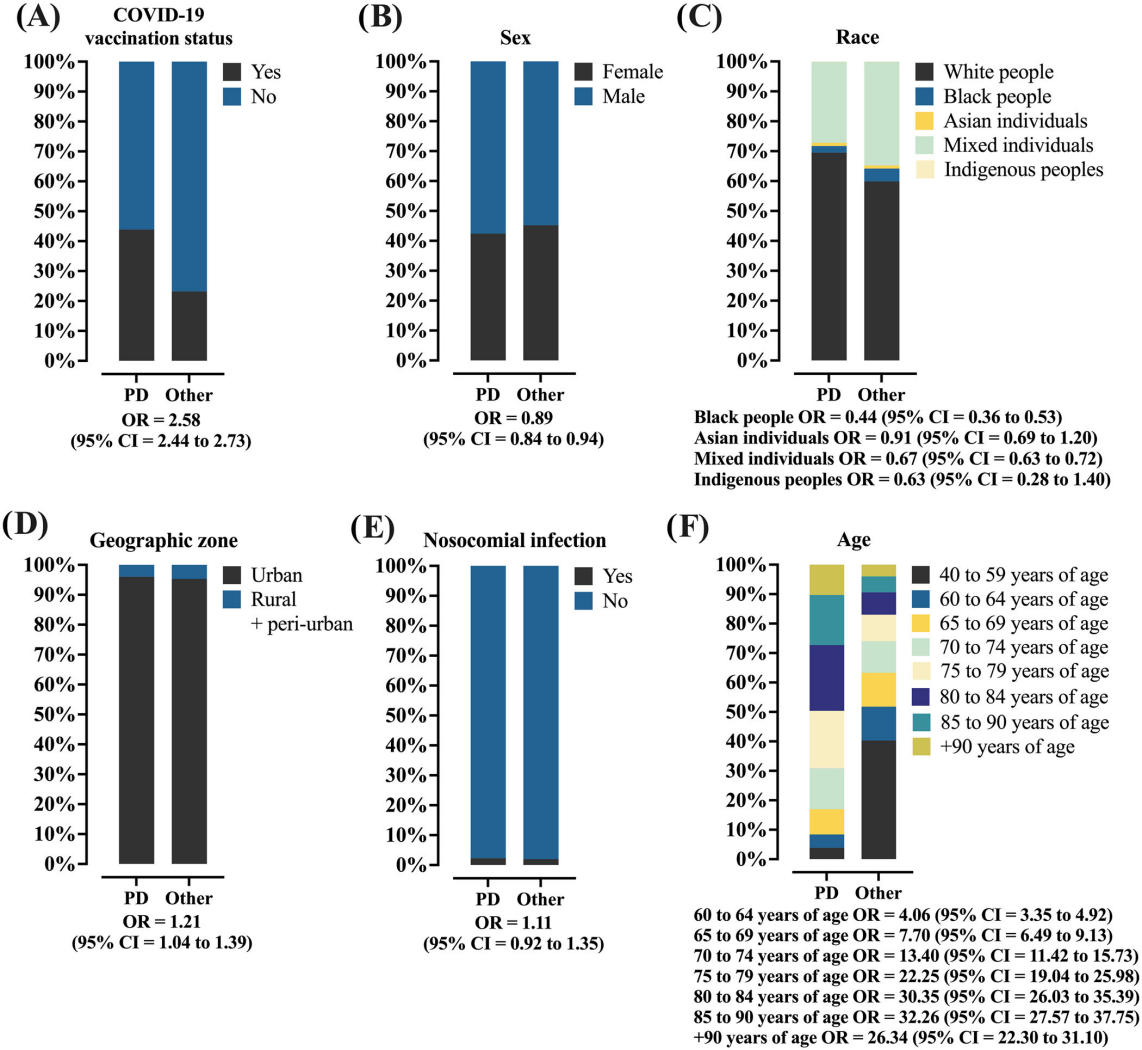
Demographic characteristics (sex, race, geographic zone of residence, and age), coronavirus disease (COVID)‐19 vaccination status, and presence of nosocomial infection of individuals hospitalized due to severe acute respiratory syndrome coronavirus 2 (SARS‐CoV‐2) infection in Brazil, according to the presence of Parkinson's disease. (A) COVID‐19 vaccination status—Distribution of vaccinated versus unvaccinated individuals according to Parkinson's disease status. (B) Sex—Proportion of male and female patients with and without Parkinson's disease. (C) Race—Distribution of racial categories (White, Black, Asian, Mixed, and Indigenous) among patients with and without Parkinson's disease. (D) Geographic zone—Distribution of patients according to residence in urban versus peri‐urban + rural areas, stratified by Parkinson's disease status. (E) Nosocomial infection—Prevalence of hospital‐acquired SARS‐CoV‐2 infection in patients with and without Parkinson's disease. (F) Age—Distribution of patients across age groups by Parkinson's disease status. Mixed: Individuals with a multiracial background (*Pardos*). Data are presented as relative frequencies (%, percentage). The association between markers was assessed using the odds ratio (OR) and the 95% confidence interval (95% CI). Statistical analysis was performed using logistic regression. A *p* value of 0.05 was considered statistically significant. The epidemiological analysis was based on data from Open‐Data‐SUS (https://opendatasus.saude.gov.br/), covering a 4‐year period of the COVID‐19 pandemic in Brazil (from February 22, 2020 to May 24, 2024). PD: patients with Parkinson's disease.

The demographic profile of patients diagnosed with Parkinson's disease was compared to that observed in the general population included in the study, as shown in Table S2. The comparison between patients with and without Parkinson's disease showed that, in general, the former were mostly male [57.6% vs. 54.8%; OR = 0.891 (95% CI = 0.842–0.943)] (Figure 1B), living in urban areas [96.0% vs. 95.3%; OR = 1.206 (95% CI = 1.044–1.392)] (Figure 1D), and self‐declared White (69.5% vs. 59.9%) (Figure 1C). This resulted in ORs lower than 1 for the other self‐declared racial categories: Black (2.2% vs. 4.3%), Mixed (27.1% vs. 34.7%), and Indigenous (0.1% vs. 0.2%). In addition, individuals diagnosed with Parkinson's disease were more likely to have received vaccination against SARS‐CoV‐2 [43.9% vs. 23.2%; OR = 2.581 (95% CI = 2.439–2.731)] (Figure 1A).

The age distribution of patients diagnosed with Parkinson's disease shows a progressive increase in prevalence with advancing age, when compared to the general population (Figure 1F). In the 40–59 age group, the proportion of patients with Parkinson's was substantially lower [3.9% vs. 40.3%], while there was a significant increase in the more advanced age groups: 60–64 [4.5% vs. 11.5%; OR = 4.059 (95% CI = 3.345–4.924)], 65–69 [8.6% vs. 11.5%; OR = 7.701 (95% CI = 6.493–9.134)], 70–74 years [13.9% vs. 10.7%; OR = 13.401 (95% CI = 11.417–15.730)], 75–79 years [19.5% vs. 9.0%; OR = 22.247 (95% CI = 19.041–25.980)], and 80–84 years [22.3% vs. 7.6%; OR = 30.352 (95% CI = 26.034–35.386)]. From the age of 85 onward, there was a slight proportional reduction, although still with high ORs: 85–90 years [17.0% vs. 5.4%; OR = 32.264 (95% CI = 27.574–37.750)] and over 90 years [10.3% vs. 4.0%; OR = 26.337 (95% CI = 22.302–31.102)].

**Figure 2:**
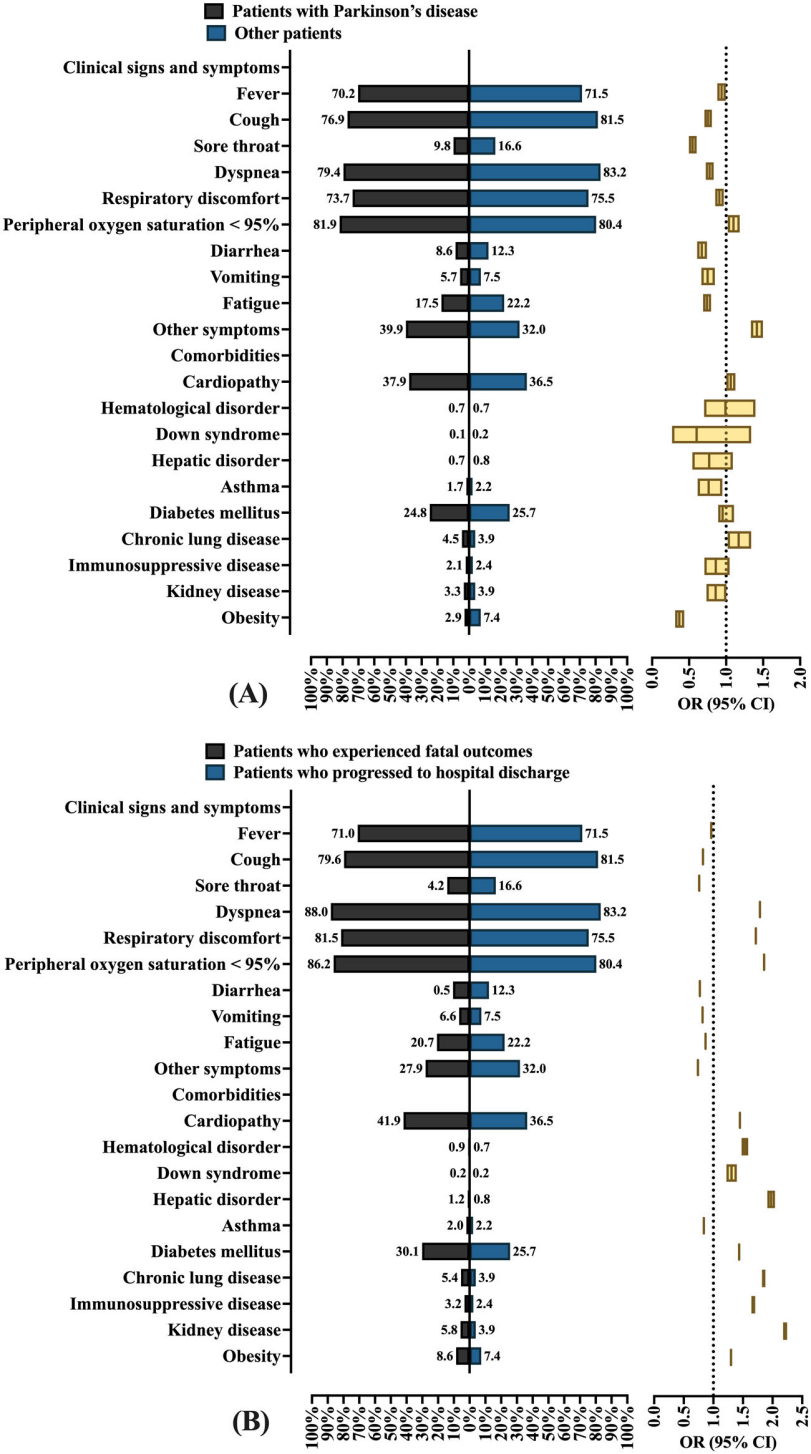
Clinical signs, symptoms, and comorbidities of hospitalized individuals due to severe acute respiratory syndrome coronavirus 2 (SARS‐CoV‐2) in Brazil. (A) Clinical signs and symptoms, and comorbidities of individuals hospitalized due to SARS‐CoV‐2 infection in Brazil, according to the presence of Parkinson's disease. (B) Clinical signs and symptoms, and comorbidities of individuals hospitalized due to SARS‐CoV‐2 infection in Brazil who evolve to death or hospital discharge (clinical recovery). Mixed: Individuals with a multiracial background (*Pardos*). Data are presented as relative frequencies (%, percentage). The association between markers was assessed using the odds ratio (OR) and the 95% confidence interval (95% CI). Statistical analysis was performed using logistic regression. A *p* value of 0.05 was considered statistically significant. The epidemiological analysis was based on data from Open‐Data‐SUS (https://opendatasus.saude.gov.br/), covering a 4‐year period of the coronavirus disease (COVID)‐19 pandemic in Brazil (from February 22, 2020 to May 24, 2024).

Patients diagnosed with Parkinson's disease were less likely to have clinical signs and symptoms on admission, with a lower prevalence of the main manifestations assessed (Figure 2A): fever (70.2% vs. 71.5%), cough (76.9% vs. 81.5%), sore throat (9.8% vs. 16.6%), dyspnea (79.4% vs. 83.2%), respiratory discomfort (73.7% vs. 75.5%), diarrhea (8.6% vs. 12.3%), vomiting (5.7% vs. 7.5%), and fatigue (17.5% vs. 22.2%). The exception was the variable peripheral oxygen saturation < 95%, which had a slightly higher frequency among patients diagnosed with Parkinson's disease [81.9% vs. 80.4%; OR = 1.100 (95% CI = 1.023–1.183]. Patients diagnosed with Parkinson's disease were less likely to have asthma [1.4% vs. 2.2%; OR = 0.764 (95% CI = 0.616–0.949) and more likely to have heart disease (cardiopathy) [37.9% vs. 36.5%; OR = 1.063 (95% CI = 1.063–1.126)] and chronic respiratory disease [4.5% vs. 3.9%; OR = 1.169 (95% CI = 1.021–1.338)] (Figure 2A).

**Figure 3:**
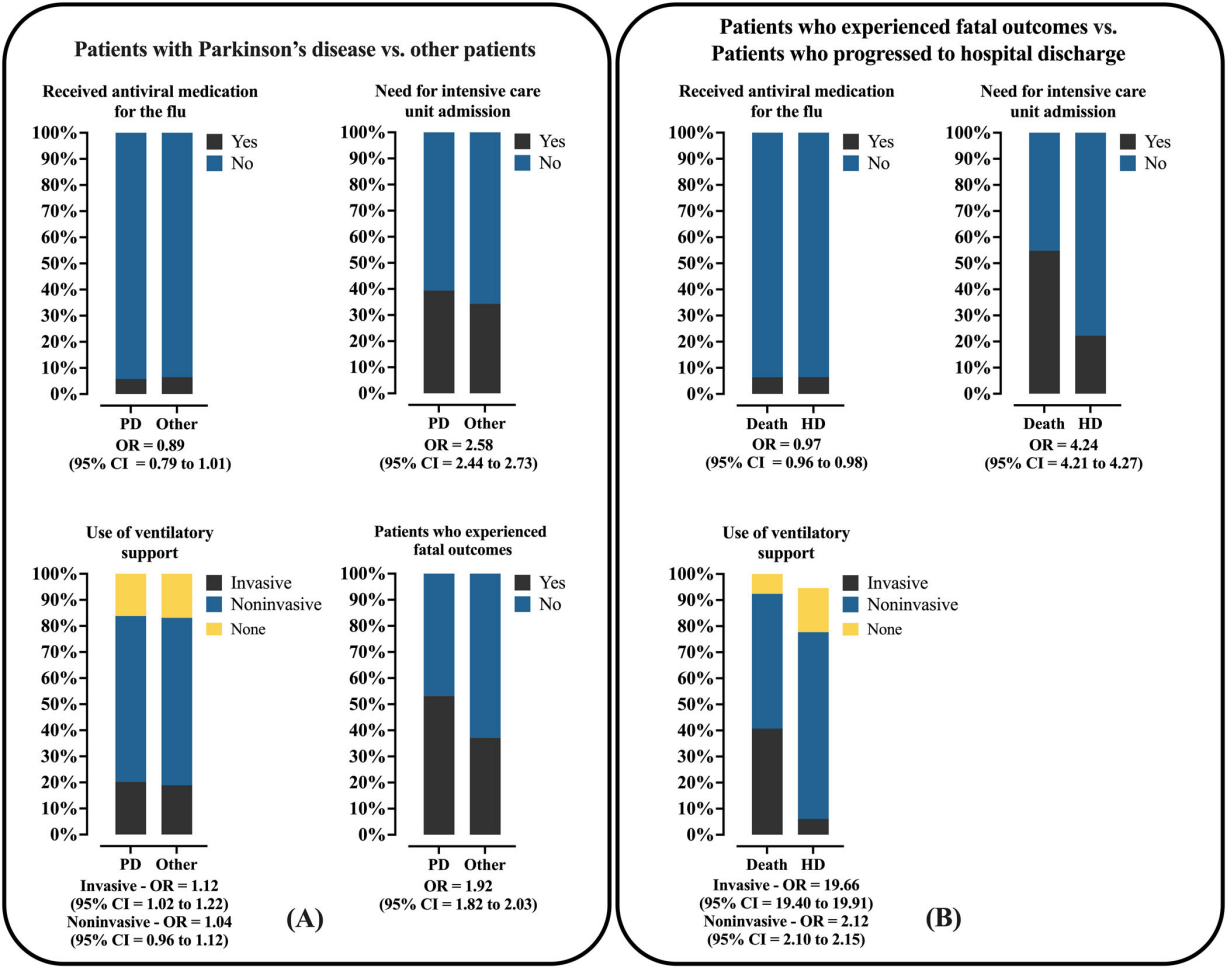
Use of antiviral medication and hospitalization data among severe acute respiratory syndrome coronavirus 2 (SARS‐CoV‐2) hospitalized patients in Brazil. (A) Use of antiviral medication to treat the flu‐like symptoms and hospitalization data of individuals hospitalized due to SARS‐CoV‐2 infection in Brazil, according to the presence of Parkinson's disease. (B) Use of antiviral medication to treat the flu‐like symptoms and hospitalization data of individuals hospitalized due to SARS‐CoV‐2 infection in Brazil who evolve to death or hospital discharge (clinical recovery). Mixed individuals: Individuals with a multiracial background (*Pardos*). Data are presented as relative frequencies (%, percentage). The association between markers was assessed using the odds ratio (OR) and the 95% confidence interval (95% CI). Statistical analysis was performed using logistic regression. A *p* value of 0.05 was considered statistically significant. The epidemiological analysis was based on data from Open‐Data‐SUS (https://opendatasus.saude.gov.br/), covering a 4‐year period of the coronavirus disease (COVID)‐19 pandemic in Brazil (from February 22, 2020 to May 24, 2024). HD, hospital discharge; PD, patients with Parkinson's disease.

There were no statistically significant differences between the groups in terms of the occurrence of nosocomial infection. Among patients diagnosed with Parkinson's disease, the need for intensive care unit admission was more frequent compared to other hospitalized patients [39.4% vs. 34.3%; OR = 1.247 (95% CI = 1.177–1.320)] (Figure 3A), as was the need for invasive ventilatory support [20.2% vs. 18.9%; OR = 1.115 (95% CI = 1.015–1.224)] (Figure 3A). Finally, there was a higher proportion of deaths among patients with Parkinson's disease compared to the general population hospitalized for COVID‐19 [53.1% vs. 37.1%; OR = 1.920 (95% CI = 1.815–2.031)] (Figure 3A).

**Figure 4:**
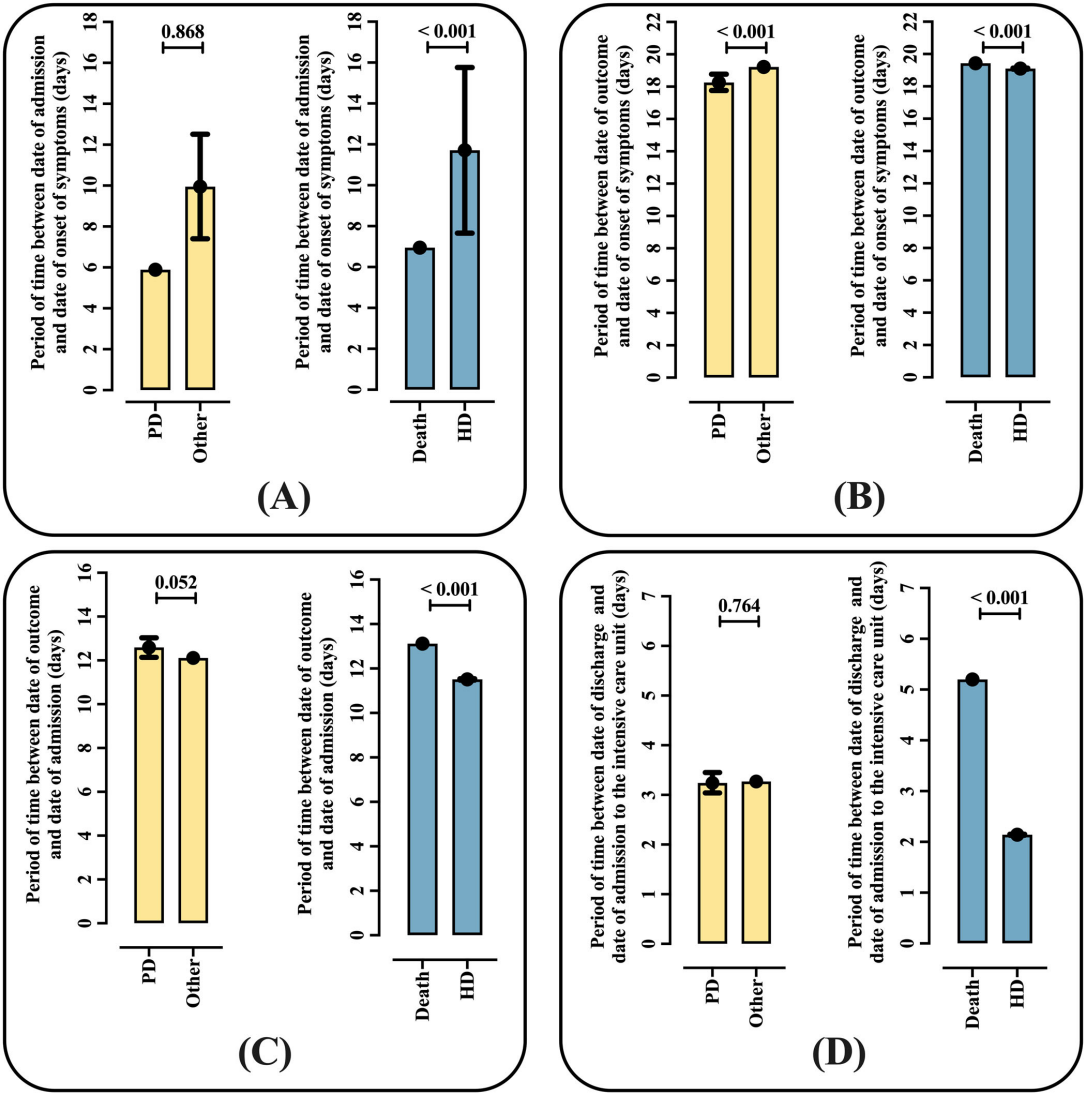
Association between the time periods related to symptoms onset, hospitalization, clinical outcome, and length of stay in the intensive care unit with the diagnosis of Parkinson's disease and patient outcomes among individuals hospitalized due to severe acute respiratory syndrome coronavirus 2 (SARS‐CoV‐2) infection during the 4‐year coronavirus disease (COVID)‐19 pandemic period in Brazil. (A) Period of time between date of admission and date of onset of symptoms. (B) Period of time between date of outcome and date of onset of symptoms. (C) Period of time between date of outcome and date of admission. (D) Period of time between date of discharge and date of admission to the intensive care unit. Data are presented as mean values with the corresponding 95% confidence interval (95% CI). Statistical analysis was performed using Student's *t*‐test. A *p* value of 0.05 was considered statistically significant. The epidemiological analysis was based on data from Open‐Data‐SUS (https://opendatasus.saude.gov.br/), covering a 4‐year period of the coronavirus disease (COVID)‐19 pandemic in Brazil (from February 22, 2020 to May 24, 2024). HD, hospital discharge; PD, patients with Parkinson's disease.

Patients diagnosed with Parkinson's disease had significantly shorter mean intervals, in days, only between the date of symptom onset and the date of the outcome (Figure 4B). The other intervals evaluated—between the date of hospital admission and the onset of symptoms (Figure 4A), between the date of hospital admission and the date of the outcome (Figure 4C), and between admission to the intensive care unit and the outcome (Figure 4D)—showed no statistically significant differences. The full data are detailed in Table S3.

**Figure 5:**
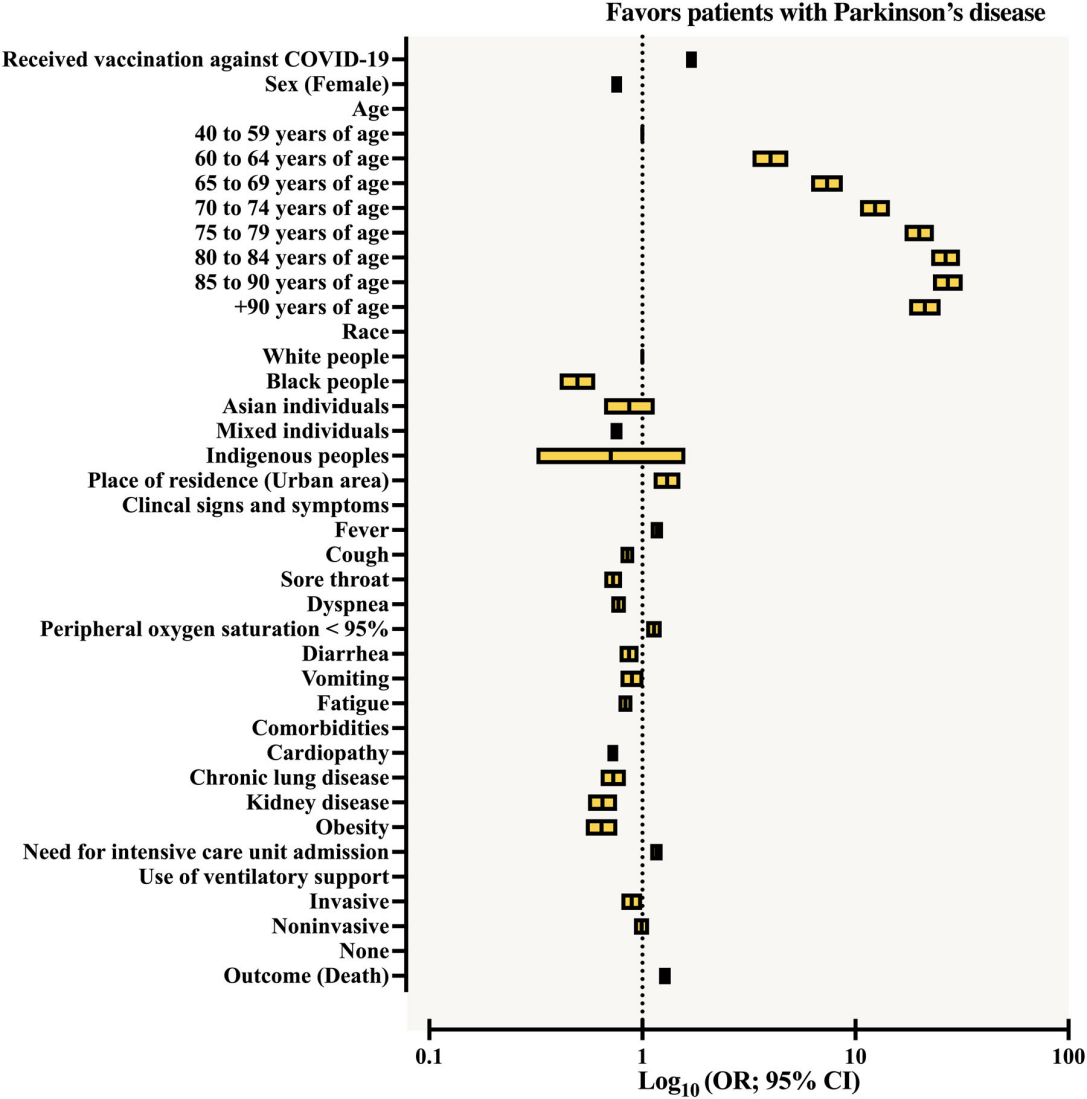
Multivariable analysis to determine the main predictors of Parkinson's disease diagnosis among those who were hospitalized due to severe acute respiratory syndrome coronavirus 2 (SARS‐CoV‐2) infection in Brazil during the 4‐year period of the pandemic. Mixed individuals: Mixed refers to individuals with a multiracial background (*Pardos*). The association between markers is described using the odds ratio (OR) and the 95% confidence interval (95% CI). Multivariable analysis was performed using the Binary Logistic Regression Model with the Backward Stepwise method. Markers with a *p* value ≤ 0.05 in the bivariate analysis were included in the regression model. The response variable was the Parkinson's disease diagnosis. Data for clinical signs and symptoms (others) and patient characteristics with a *p* value > 0.05 were excluded from the analysis. A *p* value of. 0.05 was considered statistically significant. The epidemiological analysis was based on data from Open‐Data‐SUS (https://opendatasus.saude.gov.br/), covering a 4‐year period of the coronavirus disease (COVID)‐19 pandemic in Brazil (from February 22, 2020 to May 24, 2024). Markers included in Step 1 of the multivariable analysis were: COVID‐19 vaccination status, sex, age group, race, place of residence, clinical signs and symptoms (fever, cough, sore throat, dyspnea, respiratory discomfort, peripheral oxygen saturation < 95%, diarrhea, vomiting, and fatigue), comorbidities [heart disease (cardiopathy), asthma, chronic lung disease, and obesity], need for intensive care unit admission, use of ventilatory support, outcome, and time intervals between outcome and symptom onset.

The findings obtained in the bivariate analysis were confirmed in the multivariable analysis (Table S4, Figure 5). In general, patients diagnosed with Parkinson's disease had a higher chance of death [OR = 1.277 (95% CI = 1.198–1.360)]. In addition, this group was characterized by a lower prevalence of men, a higher proportion of self‐declared Whites, older age, a greater likelihood of having received the COVID‐19 vaccine, and living in urban areas. On the other hand, there was a lower chance of presenting clinical signs and symptoms typical of COVID‐19, as well as other comorbidities. Parkinson's patients were also more likely to be admitted to an intensive care unit, but less likely to use invasive mechanical ventilation.

Diagnostic performance varied across demographic, clinical, and epidemiological markers. Age showed the highest discriminatory capacity, with sensitivity and specificity increasing progressively in older age groups, reaching values above 80% for patients aged 75–84 years. Race also contributed modestly, with higher specificity among non‐White groups. Clinical symptoms such as dyspnea and low oxygen saturation demonstrated moderate sensitivity but limited specificity. Most comorbidities and demographic variables exhibited high negative predictive values, indicating a strong capacity to rule out Parkinson's disease when absent, although positive predictive values remained low across markers (Table 3).

**Table 3:** Diagnostic performance metrics of clinical and demographic characteristics in relation to Parkinson's disease diagnosis and clinical outcomes among hospitalized patients with severe acute respiratory syndrome coronavirus 2 (SARS‐CoV‐2) infection.

Markers	Sensitivity (%)	Specificity (%)	Positive predictive value (%)	Negative predictive value (%)	Accuracy (%)
Parkinson's disease diagnosis					
Vaccination against coronavirus disease (COVID‐19)	43.86 (42.46–45.26)	76.77 (76.70–76.83)	0.54 (0.52–0.55)	99.79 (99.79–99.80)	76.67 (76.61–76.74)
Sex (male)	56.63 (56.23–59.02)	45.20 (45.13–45.28)	0.30 (0.29–0.31)	99.73 (99.72–99.74)	45.24 (45.17–45.31)
Age					
40–59 years of age (reference)	Reference	Reference	Reference	Reference	Reference
60–64 years of age	53.62 (48.69–58.51)	77.83 (77.74–77.91)	0.11 (0.10–0.12)	99.97 (99.97–99.98)	77.82 (77.73–77.90)
65–69 years of age	68.78 (64.95–72.43)	77.76 (77.67–77.84)	0.21 (0.20–0.22)	99.97 (99.97–99.98)	77.75 (77.66–77.84)
70–74 years of age	77.98 (75.08–80.69)	79.10 (79.01–79.18)	0.37 (0.36–0.38)	99.97 (99.97–99.98)	79.09 (79.01–79.18)
75–79 years of age	83.28 (80.99–85.39)	81.71 (81.63–81.79)	0.61 (0.60–0.63)	99.97 (99.97–99.98)	81.71 (81.63–81.80)
80–84 years of age	85.08 (83.02–86.99)	84.18 (84.10–84.26)	0.83 (0.81–0.85)	99.97 (99.97–99.98)	84.18 (84.10–84.26)
85–90 years of age	81.27 (78.74–83.61)	88.15 (88.08–88.22)	0.89 (0.86–0.91)	99.97 (99.97–99.98)	88.14 (88.07–88.21)
+90 years of age	72.49 (69.02–75.78)	90.90 (90.84–90.97)	0.72 (0.69–0.76)	99.97 (99.97–99.98)	90.89 (90.82–90.95)
Race					
White people (reference)	Reference	Reference	Reference	Reference	Reference
Black people	3.04 (2.50–3.66)	93.29 (93.25–93.34)	0.14 (0.12–0.17)	99.67 (99.67–99.67)	93.01 (92.96–93.05)
Asian individuals	1.50 (1.12–1.96)	98.36 (98.33–98.38)	0.30 (0.23–0.39)	99.67 (99.67–99.67)	98.04 (98.01–98.06)
Mixed individuals^a^	28.05 (26.77–29.35)	63.34 (63.26–63.41)	0.22 (0.21–0.23)	99.67 (99.66–99.68)	63.23 (63.16–63.31)
Indigenous peoples	0.18 (0.06–0.38)	99.72 (99.71–99.73)	0.21 (0.09–0.46)	99.67 (99.67–99.67)	99.39 (99.38–99.41)
Geographic zone (urban)	96.05 (95.46–96.57)	4.73 (4.70–4.76)	0.29 (0.29–0.29)	99.76 (99.73–99.79)	4.99 (4.96–5.02)
Nosocomial infection	2.26 (1.86–2.72)	97.97 (97.94–97.99)	0.32 (0.26–0.38)	99.72 (99.72–99.72)	97.69 (97.67–97.72)
Clinical signs and symptoms					
Fever	70.17 (68.86–71.44)	28.50 (28.44–28.57)	0.28 (0.27–0.28)	99.70 (99.69–99.71)	28.62 (28.55–28.69)
Cough	76.91 (75.71–78.08)	18.49 (18.44–18.55)	0.27 (0.26–0.27)	99.65 (99.63–99.66)	18.66 (18.60–18.72)
Sore throat	9.78 (8.96–10.65)	83.45 (83.39–83.50)	0.17 (0.15–0.18)	99.69 (99.69–99.70)	83.24 (83.18–83.29)
Dyspnea	79.40 (78.24–80.52)	16.79 (16.73–16.85)	0.27 (0.27–0.28)	99.65 (99.63–99.67)	16.97 (16.91–17.02)
Respiratory discomfort	73.73 (72.48–74.96)	24.46 (24.39–24.52)	0.28 (0.27–0.28)	99.69 (99.68–99.71)	24.60 (24.53–24.66)
Oxygen saturation < 95%	81.86 (80.76–82.93)	19.59 (19.54–19.65)	0.29 (0.29–0.29)	99.74 (99.72–99.75)	19.77 (19.71–19.83)
Diarrhea	8.58 (7.81–9.40)	87.74 (87.69–87.79)	0.20 (0.18–0.22)	99.70 (99.70–99.71)	87.52 (87.47–87.57)
Vomiting	5.73 (5.09–6.41)	92.52 (92.48–92.56)	0.22 (0.19–0.24)	99.71 (99.71–99.71)	92.27 (92.23–92.31)
Fatigue	17.49 (16.43–18.58)	77.77 (77.71–77.84)	0.22 (0.21–0.24)	99.70 (99.69–99.70)	77.60 (77.54–77.66)
Other symptoms	39.94 (38.57–41.33)	68.03 (67.96–68.10)	0.35 (0.34–0.37)	99.75 (99.74–99.75)	67.95 (67.88–68.02)
Comorbidities					
Heart disease (cardiopathy)	37.88 (36.52–39.26)	63.55 (63.47–63.62)	0.30 (0.29–0.31)	99.72 (99.72–99.73)	63.47 (63.40–63.54)
Hematological disorder	0.67 (0.46–0.94)	99.32 (99.31–99.33)	0.28 (0.20–0.40)	99.72 (99.71–99.72)	99.04 (99.03–99.06)
Down syndrome	0.12 (0.04–0.27)	99.80 (99.79–99.80)	0.17 (0.08–0.38)	99.72 (99.72–99.72)	99.51 (99.50–99.52)
Hepatic disorder	0.65 (0.45–0.92)	99.16 (99.14–99.17)	0.22 (0.16–0.31)	99.72 (99.71–99.72)	98.88 (98.86–98.89)
Asthma	1.71 (1.37–2.12)	97.77 (97.75–97.79)	0.22 (0.18–0.27)	99.71 (99.71–99.72)	97.50 (97.48–97.52)
Diabetes mellitus	24.80 (23.60–26.03)	74.30 (74.23–74.36)	0.27 (0.26–0.29)	99.71 (99.71–99.72)	74.16 (74.09–74.22)
Chronic respiratory disease	4.48 (3.92–5.10)	96.14 (96.11–96.17)	0.33 (0.29–0.38)	99.72 (99.72–99.72)	95.88 (95.85–95.91)
Immunosuppression disorder	2.08 (1.70–2.52)	97.59 (97.57–97.62)	0.25 (0.20–0.30)	99.71 (99.71–99.72)	97.32 (97.30–97.35)
Kidney disease	3.34 (2.86–3.88)	96.13 (96.10–96.16)	0.25 (0.21–0.29)	99.71 (99.71–99.72)	95.86 (95.83–95.89)
Obesity	2.87 (2.42–3.38)	92.56 (92.52–92.60)	0.11 (0.09–0.13)	99.70 (99.70–99.70)	92.30 (92.26–92.34)
Received antiviral medication for the flu	2.26 (1.86–2.72)	97.97 (97.94–97.99)	0.32 (0.26–0.38)	99.72 (99.72–99.72)	97.69 (97.67–97.72)
Need for intensive care unit	34.41 (38.04–40.80)	65.71 (65.64–65.78)	0.33 (0.32–0.34)	99.74 (99.73–99.74)	65.64 (65.57–65.71)
Need to mechanical ventilatory support					
Invasive	55.57 (53.23–57.90)	47.12 (47.00–47.25)	0.30 (0.29–0.32)	99.73 (99.71–99.74)	47.15 (47.02–47.27)
Noninvasive	79.74 (78.45–80.99)	20.82 (20.76–20.89)	0.28 (0.28–0.29)	99.73 (99.71–99.74)	20.99 (20.92–21.06)
No	Reference	Reference	Reference	Reference	Reference
Outcome (death)	53.16 (51.72–54.53)	62.88 (62.81–62.95)	0.41 (0.40–0.42)	99.79 (99.78–99.79)	62.85 (62.78–62.92)
Clinical outcome					
Parkinson's disease	0.41 (0.39–0.42)	99.79 (99.78–99.80)	53.13 (51.73–54.52)	62.88 (62.88–62.88)	62.85 (62.78–62.92)
Vaccination against COVID‐19	21.04 (20.94–21.14)	75.38 (75.30–75.46)	37.17 (37.09–37.24)	33.57 (33.44–33.70)	55.18 (55.11–55.26)
Sex (male)	55.68 (55.56–55.81)	45.72 (45.62–45.81)	37.76 (37.70–37.83)	63.56 (63.48–63.64)	49.42 (49.35–49.50)
Age					
40–59 years of age (reference)	Reference	Reference	Reference	Reference	Reference
60–64 years of age	29.95 (29.76–30.13)	80.61 (80.51–80.71)	35.78 (35.59–35.96)	76.14 (76.08–76.19)	67.18 (67.08–67.28)
65–69 years of age	33.16 (32.97–33.34)	81.90 (81.81–82.00)	41.30 (41.12–41.49)	76.14 (76.08–76.19)	68.38 (68.28–68.47)
70–74 years of age	33.66 (33.47–33.84)	84.08 (83.99–84.17)	45.65 (45.45–45.85)	76.14 (76.08–76.19)	69.75 (69.65–69.84)
75–79 years of age	31.70 (31.52–31.89)	86.94 (86.86–87.03)	49.20 (48.98–49.42)	76.14 (76.08–76.19)	71.19 (71.09–71.28)
80–84 years of age	29.35 (29.17–29.54)	89.40 (89.32–89.48)	52.34 (52.09–52.58)	76.14 (76.09–76.19)	72.35 (72.25–72.44)
85–90 years of age	23.84 (23.66–24.01)	92.59 (92.52–92.66)	55.06 (54.77–55.35)	76.14 (76.09–76.18)	73.62 (73.52–73.72)
+90 years of age	20.08 (19.91–20.26)	94.91 (94.86–94.97)	59.51 (59.17–59.85)	76.14 (76.10–76.18)	74.61 (74.52–74.71)
Race					
White people (reference)	Reference	Reference	Reference	Reference	Reference
Black people	8.15 (8.07–8.24)	94.12 (94.06–94.17)	43.59 (43.24–43.93)	64.75 (64.73–64.78)	63.33 (63.24–63.42)
Asian individuals	1.67 (1.63–1.71)	98.37 (98.34–98.40)	35.84 (35.13–36.55)	64.75 (64.74–64.76)	64.28 (64.18–64.37)
Mixed individuals^a^	39.43 (39.31–39.56)	65.00 (64.90–65.09)	39.69 (39.59–39.49)	64.75 (64.69–64.81)	55.57 (55.49–55.65)
Indigenous peoples	0.34 (0.33–0.36)	99.76 (99.74–99.77)	43.56 (41.76–45.37)	64.75 (64.75–64.76)	64.69 (64.60–64.78)
Geographic zone (urban)	94.91 (94.86–94.96)	4.51 (4.47–4.55)	37.02 (37.01–37.04)	59.97 (59.64–60.30)	38.11 (38.04–38.18)
Nosocomial infection	2.59 (2.55–2.63)	98.29 (98.27–98.32)	47.30 (46.78–47.81)	63.05 (63.03–63.06)	62.72 (62.65–62.80)
Clinical signs and symptoms					
Fever	71.02 (70.91–71.13)	28.23 (28.14–28.31)	36.92 (36.88–36.97)	62.22 (62.11–62.34)	44.13 (44.06–44.21)
Cough	79.64 (79.54–79.74)	17.41 (17.34–17.48)	36.32 (36.28–36.35)	59.11 (58.96–59.26)	40.54 (40.46–40.61)
Sore throat	14.23 (14.14–14.31)	82.10 (82.03–82.17)	31.98 (31.82–32.14)	61.81 (61.78–61.84)	56.88 (56.80–56.95)
Dyspnea	87.98 (87.90–88.06)	19.63 (19.55–19.70)	39.30 (39.27–39.33)	73.40 (73.25–73.55)	45.03 (44.95–45.10)
Respiratory discomfort	81.54 (81.44–81.63)	28.01 (27.93–28.10)	40.12 (40.08–40.16)	71.95 (71.83–72.07)	47.90 (47.83–47.98)
Oxygen saturation < 95%	86.15 (86.07–86.24)	22.99 (22.91–23.07)	37.17 (37.09–37.24)	73.73 (73.59–73.87)	46.46 (46.39–46.54)
Diarrhea	10.55 (10.47–10.62)	86.75 (86.68–86.81)	32.00 (31.82–32.19)	62.11 (62.09–62.14)	58.43 (58.35–58.50)
Vomiting	6.62 (6.56–6.68)	92.02 (91.97–92.07)	32.93 (32.68–33.18)	62.49 (62.47–62.51)	60.28 (60.21–60.36)
Fatigue	20.68 (20.58–20.77)	76.88 (76.80–76.96)	34.59 (34.46–34.73)	62.10 (62.06–62.14)	55.99 (55.92–56.06)
Other symptoms	27.91 (27.80–28.02)	65.59 (65.50–65.68)	32.42 (32.32–32.52)	60.60 (60.55–60.65)	51.59 (51.51–51.66)
Comorbidities					
Heart disease (cardiopathy)	41.90 (41.78–42.02)	66.76 (66.67–66.85)	42.72 (42.62–42.81)	66.02 (65.96–66.08)	57.52 (57.45–57.60)
Hematological disorder	0.87 (0.84–0.89)	99.43 (99.42–99.45)	47.46 (46.56–48.36)	62.91 (62.90–62.91)	62.80 (62.73–62.87)
Down syndrome	0.24 (0.23–0.25)	99.82 (99.81–99.83)	43.57 (41.94–45.22)	62.85 (62.84–62.85)	62.81 (62.74–62.88)
Hepatic disorder	1.22 (1.19–1.25)	99.38 (99.36–99.39)	53.72 (52.91–54.52)	62.98 (62.97–62.98)	62.90 (62.83–62.97)
Asthma	1.99 (1.96–2.03)	97.64 (97.61–97.66)	33.28 (32.82–33.75)	62.75 (62.74–62.76)	62.09 (62.02–62.16)
Diabetes mellitus	30.11 (30.00–30.22)	76.91 (76.83–76.99)	43.54 (43.42–43.67)	65.04 (65.00–65.08)	59.52 (59.44–59.59)
Chronic respiratory disease	5.37 (5.31–5.42)	97.03 (96.99–97.06)	51.63 (51.26–52.01)	63.42 (63.40–63.43)	62.96 (62.89–63.03)
Immunosuppression disorder	3.20 (3.16–3.24)	98.06 (98.04–98.09)	49.43 (48.95–49.90)	63.14 (63.12–63.15)	62.81 (62.74–62.88)
Kidney disease	5.82 (5.76–5.88)	97.28 (97.25–97.31)	55.87 (55.50–56.24)	63.59 (63.57–63.60)	63.29 (63.22–63.36)
Obesity	8.58 (8.51–8.65)	93.25 (93.21–93.30)	42.94 (42.68–43.20)	63.30 (63.28–63.32)	61.79 (61.71–61.86)
Received antiviral medication for the flu	6.36 (6.30–6.42)	93.47 (93.42–93.52)	36.55 (36.28–36.82)	62.79 (62.77–62.81)	61.10 (61.02–61.17)
Need for intensive care unit	54.76 (54.64–55.88)	77.80 (77.72–77.88)	59.33 (59.23–59.43)	74.41 (74.35–74.46)	69.24 (69.17–69.30)
Need to mechanical ventilatory support					
Invasive	84.25 (84.12–84.38)	78.61 (78.46–78.75)	79.82 (79.71–79.93)	83.25 (83.13–83.37)	81.43 (81.34–81.53)
Noninvasive	87.18 (87.07–87.28)	23.81 (23.73–23.89)	29.94 (29.91–29.98)	83.25 (83.13–83.38)	41.04 (40.96–41.12)
No	Reference	Reference	Reference	Reference	Reference

Abbreviation: %, percentage. The data are presented as percentages and 95% confidence intervals.

^a^ Individuals with a multiracial background (*Pardos*).

The logistic regression model for distinguishing patients with Parkinson's disease also showed good discrimination, with an AUC of 0.808 (95% CI = 0.803–0.813), reflecting robust ability to differentiate between patients with and without the condition.

### 3.3 Factors Associated With Death in Patients Hospitalized for SARS‐CoV‐2 Infection

**Figure 6:**
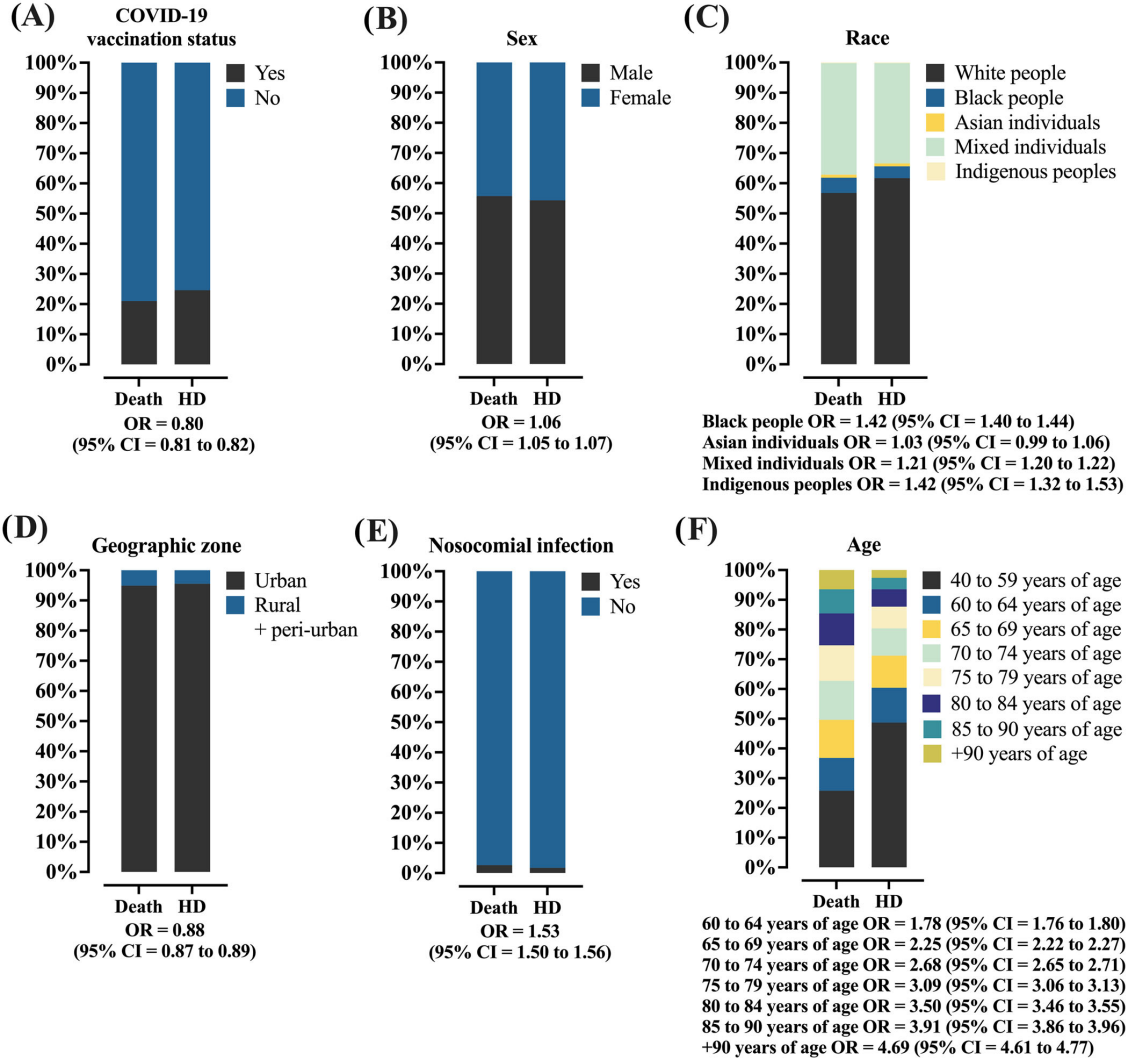
Demographic characteristics (sex, race, geographic zone of residence, and age), coronavirus disease (COVID)‐19 vaccination status, and presence of nosocomial infection of individuals hospitalized due to severe acute respiratory syndrome coronavirus 2 (SARS‐CoV‐2) infection in Brazil who evolve for death or hospital discharge (clinical recovery). (A) COVID‐19 vaccination status—Distribution of vaccinated versus unvaccinated individuals according to clinical outcome (death or hospital discharge). (B) Sex—Proportion of male and female patients according to clinical outcome (death or hospital discharge). (C) Race—Distribution of racial categories (White, Black, Asian, Mixed, and Indigenous) according to clinical outcome (death or hospital discharge). (D) Geographic zone—Distribution of patients according to residence in urban versus peri‐urban + rural areas, stratified by clinical outcome (death or hospital discharge). (E) Nosocomial infection—Prevalence of hospital‐acquired SARS‐CoV‐2 infection according to clinical outcome (death or hospital discharge). (F) Age—Distribution of patients across age by according to clinical outcome (death or hospital discharge). Mixed: Individuals with a multiracial background (*Pardos*). Data are presented as relative frequencies (%, percentage). The association between markers was assessed using the odds ratio (OR) and the 95% confidence interval (95% CI). Statistical analysis was performed using logistic regression. A *p* value of 0.05 was considered statistically significant. The epidemiological analysis was based on data from Open‐Data‐SUS (https://opendatasus.saude.gov.br/), covering a 4‐year period of the COVID‐19 pandemic in Brazil (from February 22, 2020 to May 24, 2024). HD, hospital discharge.

The demographic profile of patients who died was analyzed in comparison to those who were discharged from the hospital, as detailed in Table S5. There was a predominance of males among the deaths [55.7% vs. 54.3%; OR = 1.058 (95% CI = 1.052–1.065)] (Figure 6B), while the proportion of individuals from urban areas was slightly lower in this group [94.9% vs. 95.5%; OR = 0.881 (95% CI = 0.868–0.894)] (Figure 6D). With regard to race/ethnicity, there was a lower representation of self‐declared White patients among deaths (56.8% vs. 61.7%), while the other categories showed a greater association with the risk of death: Black [5.0% vs. 3.9%; OR = 1.419 (95% CI = 1.398–1.441)], Mixed [37.0% vs. 33.2%; OR = 1.209 (95% CI = 1.201–1.217)], and Indigenous [0.2% vs. 0.2%; OR = 1.418 (95% CI = 1.317–1.526)] (Figure 6C). In addition, vaccination coverage against SARS‐CoV‐2 was lower among patients who died [21.0% vs. 24.6%; OR = 0.816 (95% CI = 0.810–0.822)] (Figure 6A).

The patients who died had a higher prevalence of various comorbidities compared to those who were discharged from hospital: heart disease (cardiopathy) (41.9% vs. 33.2%), hematological disorders (0.9% vs. 0.6%), Down syndrome (0.2% vs. 0.2%), liver disorders (1.2% vs. 0.6%), diabetes mellitus (30.1% vs. 23.1%), chronic respiratory diseases (5.4% vs. 3.0%), immunosuppressive conditions (3.2% vs. 1.9%), kidney diseases (5.8% vs. 2.7%), and obesity (8.6% vs. 6.7%). The only exception was asthma, which was less frequent among patients who died [2.0% vs. 2.4%; OR = 0.840 (95% CI = 0.822–0.858)] (Figure 2B).

With regard to clinical signs and symptoms, patients who died had a higher frequency of most of the manifestations analyzed, especially those related to the respiratory system: sore throat (14.2% vs. 17.9%), dyspnea (88.0% vs. 80.4%), respiratory discomfort (81.5% vs. 72.0%), and peripheral oxygen saturation < 95% (86.2% vs. 77.0%). Some exceptions were observed, with slightly lower prevalence rates among deaths: fever (71.0% vs. 71.8%), cough (79.6% vs. 82.6%), diarrhea (10.5% vs. 13.3%), vomiting (6.6% vs. 8.0%), and fatigue (20.7% vs. 23.1%) (Figure 2B).

The age distribution of patients who died followed the pattern previously described in the literature, showing a progressive increase in the prevalence of mortality with advancing age. Compared to the 40–59 age group [25.8% among deaths versus 40.5% among patients discharged from hospital], there was a significant increase in the risk of death in the higher age groups: 60–64 years [11.0% vs. 11.7%; OR = 1.777 (95% CI = 1.758–1.796)], 65–69 years [12.8% vs. 10.8%; OR = 2.245 (95% CI = 2.221–2.268)], 70–74 years [13.1% vs. 9.2%; OR = 2.680 (95% CI = 2.651–2.709)], 75–79 years [12.0% vs. 7.3%; OR = 3.090 (95% CI = 3.055–3.125)], 80–84 years [10.7% vs. 5.8%; OR = 3.503 (95% CI = 3.461–3.546)], 85–90 years [8.1% vs. 3.9%; OR = 3.909 (95% CI = 3.855–3.964)], and over 90 years [6.5% vs. 2.6%; OR = 4.689 (95% CI = 4.614–4.765)] (Figure 6F).

Patients who died had a higher prevalence of nosocomial infection [2.6% vs. 1.5%; OR = 1.531 (95% CI = 1.499–1.564)] (Figure 6E) and a lower propensity to use antivirals to manage flu symptoms [6.4% vs. 6.5%; OR = 0.971 (95% CI = 0.960–0.984)] (Figure 3B). However, this group was more frequently hospitalized in intensive care units [54.8% vs. 22.2%; OR = 4.241 (95% CI = 4.213–4.270)] and subjected to both invasive mechanical ventilation [40.7% vs. 6.1%; OR = 19.657 (95% CI = 19.404–19.913)] and noninvasive mechanical ventilation [51.7% vs. 71.5%; OR = 2.124 (95% CI = 2.102–2.147)], compared to those who did not require ventilatory support (7.6% vs. 22.4%) (Figure 3B).

Patients who died had significantly shorter mean time intervals, in days, between the date of hospital admission and the onset of symptoms, and longer between the date of the outcome and the onset of symptoms, between the date of the outcome and hospital admission, as well as between the date of the outcome and admission to an intensive care unit (Figure 4A–D). The full data is detailed in Table S3.

**Figure 7:**
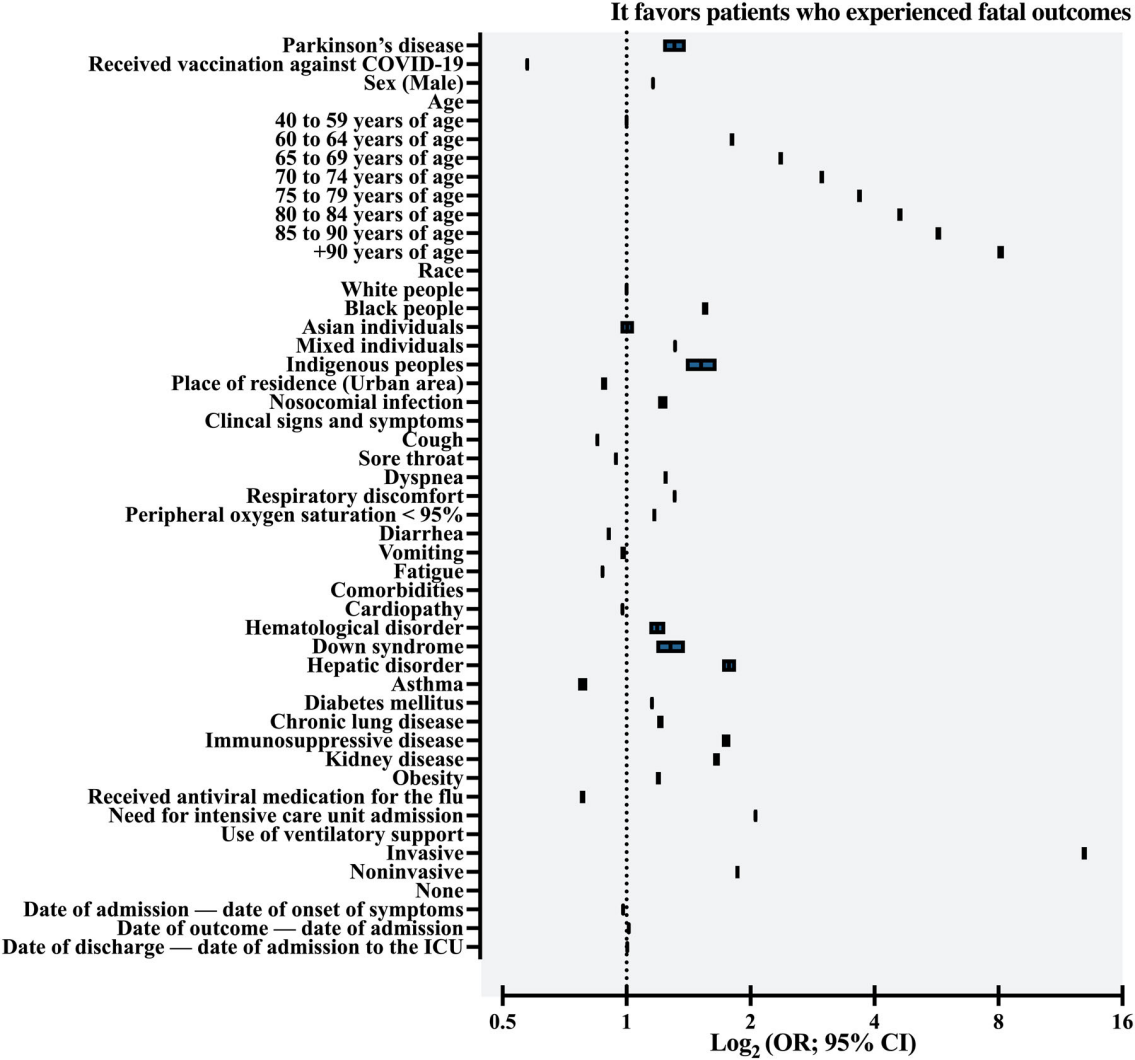
Multivariable analysis to determine the main predictors of death among those who were hospitalized due to severe acute respiratory syndrome coronavirus 2 (SARS‐CoV‐2) infection in Brazil during the 4‐year period of the pandemic. Mixed individuals: Mixed refers to individuals with a multiracial background (*Pardos*). The association between markers is described using the odds ratio (OR) and the 95% confidence interval (95% CI). Multivariable analysis was performed using the Binary Logistic Regression Model with the Backward Stepwise method. Markers with a *p* value ≤ 0.05 in the bivariate analysis were included in the regression model. The dependent variable was mortality. Data for clinical signs and symptoms (others) and patient characteristics with a *p* value > 0.05 were excluded from the analysis. A *p* value of. 0.05 was considered statistically significant. The epidemiological analysis was based on data from Open‐Data‐SUS (https://opendatasus.saude.gov.br/), covering a 4‐year period of the coronavirus disease (COVID)‐19 pandemic in Brazil (from February 22, 2020 to May 24, 2024). Markers included in step 1 of the multivariable analysis: Parkinson's disease diagnosis, COVID‐19 vaccination status, sex, age group, race, place of residence, nosocomial infection, clinical signs and symptoms (fever, cough, sore throat, dyspnea, respiratory discomfort, peripheral oxygen saturation < 95%, diarrhea, vomiting, and fatigue), comorbidities [heart disease (cardiopathy), hematological disorder, Down syndrome, hepatic disorder, asthma, diabetes mellitus, chronic lung disease, immunosuppressive disease, kidney disease, and obesity], antiviral medication for the flu‐like symptoms, need for intensive care unit admission, use of ventilatory support, time intervals between admission and symptom onset, outcome and symptom onset, outcome and admission, and discharge and intensive care unit admission.

The findings obtained in the bivariate analysis were confirmed by the multivariable analysis (Table S6, Figure 7). In general, patients diagnosed with Parkinson's disease had a higher chance of death [OR = 1.307 (95% CI = 1.226–1.393)]. The higher risk of mortality was associated with a higher prevalence of males, self‐declared race as Black, Mixed, or Indigenous, older age, lower likelihood of having received the COVID‐19 vaccine, and living in an urban area. In addition, these patients were more likely to show clinical signs and symptoms typical of COVID‐19, as well as to have comorbidities. Those who died were also more likely to have nosocomial infections, to use invasive and noninvasive mechanical ventilation, as well as shorter average intervals between the date of hospital admission and the onset of symptoms, and longer intervals between the date of the outcome and hospital admission, as well as between the date of the outcome and admission to an intensive care unit.

For mortality prediction, several markers displayed relevant diagnostic performance. Advanced age groups were associated with progressively higher specificity and positive predictive values, particularly in individuals ≥ 80 years. Male sex and lack of vaccination were also linked to worse outcomes, though with moderate accuracy. Clinical signs such as dyspnea and respiratory discomfort showed high sensitivity, while invasive mechanical ventilation demonstrated both high sensitivity and specificity. Comorbidities including diabetes mellitus, heart disease (cardiopathy), and kidney disease presented moderate diagnostic performance. Overall, the models reflected strong discriminatory ability for severe outcomes, especially when combining demographic, clinical, and comorbidity data.

The logistic regression model for mortality demonstrated good discrimination, with an AUC of 0.810 (95% CI = 0.809–0.811), indicating reliable performance in distinguishing patients who experienced the outcome.

**Figure 8:**
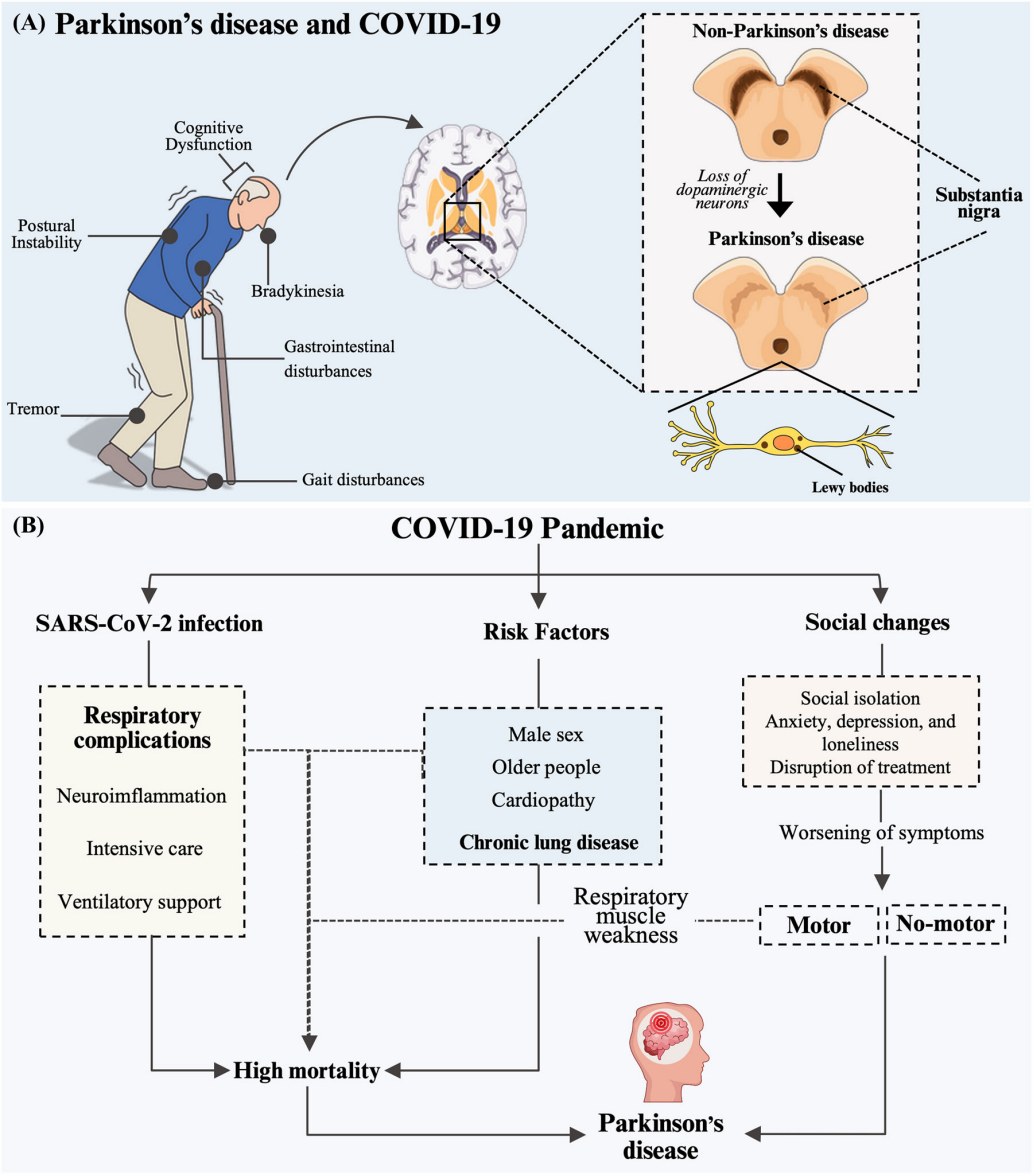
Key factors associated with severe acute respiratory syndrome coronavirus 2 (SARS‐CoV‐2) infection in individuals with Parkinson's disease and the general population, highlighting major mortality risk factors and clinical progression during the coronavirus disease (COVID‐19) pandemic. (A) Neuropathological features of Parkinson's disease: cognitive dysfunction, bradykinesia, gastrointestinal disturbances, gait impairment, tremor, and postural instability. The non‐Parkinson illustration represents a healthy brain, while the Parkinson illustration shows degeneration of the substantia nigra and accumulation of Lewy bodies, the hallmarks of Parkinson's disease. (B) Shared risk factors for SARS‐CoV‐2 infection and Parkinson's disease include male sex, advanced age, comorbid cardiovascular, and respiratory conditions—all linked to increased COVID‐19 mortality. Additional risk factors specific to SARS‐CoV‐2 infection—such as neurodegeneration, requirement for intensive care, and need for ventilatory support—further elevate mortality risk. Indirect pandemic‐related effects—social isolation, treatment disruptions, anxiety, depression, and loneliness—also exacerbate both motor and non‐motor Parkinson's symptoms.

Key factors associated with SARS‐CoV‐2 infection in individuals with Parkinson's disease and the general population, highlighting major mortality risk factors and clinical progression during the COVID‐19 pandemic, are presented in Figure 8 and Figure S1.

## 4 Discussion

In this study, which analyzed 1 725 690 SARS‐CoV‐2–related hospitalizations in Brazil among patients aged 40 years or older, there was a demographic predominance of males, self‐declared White individuals, and urban residents, especially in the Southeast region. Respiratory symptoms were the most frequent, with high prevalence of dyspnea, cough, and oxygen desaturation. Among these patients, 0.3% had a prior diagnosis of Parkinson's disease—a group distinguished by significantly older age, a higher proportion of White individuals, and higher COVID‐19 vaccination rates. Although patients with Parkinson's disease exhibited a lower frequency of the classic infection symptoms, they showed greater need for intensive care admission, increased use of invasive mechanical ventilation, and, above all, significantly higher mortality compared with the general hospitalized population. Multivariable analysis confirmed that Parkinson's disease was independently associated with an increased risk of death even after adjustment for age, sex, comorbidities, and vaccination status, underscoring the particular vulnerability of this group to SARS‐CoV‐2 infection.

### 4.1 Epidemiological Characteristics of Patients With Parkinson's Disease Hospitalized for SARS‐CoV‐2 Infection

Analysis of our data delineated the epidemiological profile of patients with Parkinson's disease hospitalized for SARS‐CoV‐2 infection: predominantly male, self‐declared White, and elderly individuals residing mainly in urban areas. These findings are consistent with previous reports—for example, Scherbaum et al. [22] in Germany, which showed a male predominance among Parkinson's disease patients hospitalized for COVID‐19, with cases beginning at age 40 and prevalence rising with advancing age.

International studies show variability in incidence and prevalence of Parkinson's disease depending on the population and age group studied. In Norway, prevalence was 0.20% in women and 0.23% in men in the general population, increasing with age [23]. German burden‐of‐disease data for 2022 report a prevalence of 0.35% in the overall population, rising to 0.61% among those over 40 years (0.57% in women; 0.66% in men) [24]. In Brazil, a cohort study estimated a crude prevalence of 0.84% among individuals aged 50 or older, with a higher proportion of males and increasing prevalence with age [25].

Epidemiological research faces methodological challenges, such as misclassification of other neurodegenerative diseases, secondary tremor disorders, and nonprogressive conditions that mimic Parkinson's disease, as well as underdiagnosis due to attributing symptoms to normal aging, limited healthcare access, or diagnostic confusion with comorbidities like osteoarthritis, frailty, and depression [26]. These diagnostic difficulties extend into daily clinical practice and highlight the urgent need to improve healthcare professionals’ training in diagnosing and managing neurodegenerative conditions. In addition, public health education must emphasize early recognition of signs and symptoms and the implementation of effective prevention strategies. Projections indicate that by 2050 approximately 25.2 million people worldwide will live with Parkinson's disease (age‐standardized prevalence of 216 cases per 100 000 population—a 55% increase since 2021) [27].

Prevalence of Parkinson's disease increases progressively with age and is higher in males than in females [28]. Our findings corroborate this pattern, showing a significant rise in the proportion of diagnoses through the 80–84‐year age band, followed by a subsequent decline, consistent with systematic‐review data that identify a prevalence peak between 85 and 89 years and confirm greater male predominance [21].

The reasons for higher prevalence among males remain incompletely understood but may involve biological factors such as differential gene expression and estrogen's neuroprotective effects in the dopaminergic system, as well as environmental and behavioral influences [29]. There is also evidence that clinical manifestations differ by sex: females tend to have later onset of motor symptoms and more non‐motor features—including gastrointestinal disturbances, loss of taste and smell, pain, sleep alterations, anxiety, and depression—and to face disparities in access to and quality of care [29, 30]. Moreover, male sex is an important risk factor for severe COVID‐19, largely due to differences in inflammatory responses. Women tend to exhibit a stronger innate and adaptive immune response to SARS‐CoV‐2 infection [31, 32, 33, 34, 35]. Zhao et al. [36] reported that immune dysregulation associated with worse prognosis in SARS‐CoV‐2–infected individuals was more pronounced in men than in women. These observations underscore the need for an individualized, sex‐specific approach to hospital management of Parkinson's disease patients.

Although most patients with Parkinson's disease in this study self‐identified as White—a pattern mirrored in other research—it is important to highlight socioeconomic and ethnoracial disparities that significantly affect healthcare access and may contribute to underdiagnosis in minority populations [26]. A United States cohort study demonstrated that minority individuals with Parkinson's disease report poorer health‐related quality of life and reduced access to specialists, medications, and nonpharmacological therapies [37]. These findings reinforce the need for public policies that promote equity in diagnosis and treatment and strategies to expand specialized care access across all demographic groups.

In our cohort, patients with Parkinson's disease hospitalized for SARS‐CoV‐2 infection had fewer comorbidities compared with other patients—except for heart diseases (cardiopathies) and respiratory diseases. Literature suggests a possible association between Parkinson's disease and cardiovascular conditions, though the underlying pathophysiological mechanisms remain unclear. Both share biological pathways—chronic inflammation, insulin resistance, lipid metabolism alterations, and oxidative stress—underscoring the need for vigilant cardiovascular monitoring in these hospitalized patients [38, 39].

Regarding respiratory changes, a narrative review identified that respiratory disorders—including obstructive and restrictive dysfunctions, central breathing control alterations, sleep apnea syndrome, and aspiration pneumonia—may occur even in early stages of Parkinson's disease, before motor symptoms appear, highlighting the importance of systematic respiratory assessment [40]. A Finnish study found that 96.7% of Parkinson's disease patients reported at least one comorbidity—most commonly systemic arterial hypertension, osteoarthritis, fractures, and prostatic hyperplasia [41]. Carroll et al. [42] further emphasize comprehensive monitoring—assessing fall risk, bone health, cardiovascular profile, anticholinergic medication burden, and sleep disorders—in this population.

Therefore, our findings should be interpreted cautiously, as they may reflect underreporting or missing documentation of relevant comorbidities in hospital information systems. Such gaps reinforce the need for a multidimensional, proactive clinical approach to the management of hospitalized patients with Parkinson's disease to ensure more effective and individualized interventions.

### 4.2 Impact of SARS‐CoV‐2 Infection in Patients With Parkinson's Disease

Patients with a prior diagnosis of Parkinson's disease were less likely upon admission to exhibit severe clinical signs; respiratory manifestations—especially oxygen desaturation, dyspnea, and cough—were most commonly observed.

A 2022 systematic review and meta‐analysis reported that fever, cough, fatigue, and anorexia were the symptoms most frequently described by Parkinson's disease patients with COVID‐19, but no significant differences in cough or fever rates were found when comparing patients with and without Parkinson's disease [13]. Diagnosis of COVID‐19 in these patients may be delayed due to symptom overlap with Parkinson's disease itself (fatigue, anosmia, myalgia, and gastrointestinal disturbances) or because the clinical picture may present atypically as an exacerbation of previously controlled motor symptoms [43].

An online observational study comparing individuals with and without Parkinson's disease found no significant differences in initial COVID‐19 symptoms; however, patients with Parkinson's disease reported worsening of their motor symptoms and emergence of new motor and non‐motor manifestations—mood disturbances, cognitive changes, sleep disruption, and loss of functional autonomy [44]. Similar results were reported by Yin et al. [45], who identified cough, fever, and fatigue as the most prevalent symptoms in Parkinson's disease patients diagnosed with COVID‐19, with over one‐third describing worsening of motor symptoms such as resting tremor, bradykinesia, and rigidity.

A study during the first 15 months of the pandemic found an increased risk of SARS‐CoV‐2 infection among individuals with Parkinson's disease and parkinsonism compared with control populations, but did not observe significant differences in hospitalization rates or lengths of stay [46]. These findings align with a 2022 systematic review and meta‐analysis that estimated a COVID‐19 prevalence of 1.06% and a hospitalization rate of 0.98% among patients with Parkinson's disease, also reporting no statistically significant differences in infection or hospitalization rates versus non‐Parkinson's cohorts [47].

In our cohort, patients with Parkinson's disease required intensive care admission and invasive mechanical ventilation more often than other hospitalized patients, suggesting a more severe clinical course. These results are consistent with data from a Shanghai hospital, in which Parkinson's disease patients more frequently needed oxygen therapy, had longer hospital stays, and experienced higher mortality [48]. Moreover, a systematic review of COVID‐19 outcomes in Parkinson's disease showed that this neurological condition is associated with worse hospital outcomes, including increased infection severity and higher mortality [49].

SARS‐CoV‐2 infection triggers a systemic inflammatory response–characterized by cytokine storm, oxidative stress, and immune dysfunction—that may upregulate α‐synuclein expression and Lewy body formation, contributing to neuroinflammation and dopaminergic neuron degeneration, thus potentially exacerbating preexisting Parkinson's disease [50]. Another interaction mechanism involves the angiotensin‐converting enzyme 2 receptor (ACE2), which mediates viral entry and is implicated in Parkinson's disease pathophysiology—renin–angiotensin system dysregulation, neuroinflammation, oxidative stress, and dopaminergic degeneration. Evidence suggests that SARS‐CoV‐2 may directly or indirectly affect the central nervous system via ACE2 interactions [51, 52].

A neuropathological comparison of individuals who died from COVID‐19 versus pneumonia or respiratory failure identified SARS‐CoV‐2–immunoreactive neurons in the dorsal medulla and substantia nigra of Parkinson's disease patients, as well as tyrosine hydroxylase–positive neurites and viral RNA in these regions, suggesting possible viral antigen presence in dopaminergic neurons [53].

Conversely, some medications commonly used by Parkinson's disease patients—amantadine, levodopa—and vitamin D supplementation may confer beneficial effects during SARS‐CoV‐2 infection [50]. In our study, higher COVID‐19 vaccination adherence was observed among Parkinson's disease patients, likely reflecting more frequent medical follow‐up. Yin et al. [45] support this, demonstrating that vaccination within 3 months before infection was associated with lower rates of SARS‐CoV‐2 infection in Parkinson's disease patients, indicating short‐term protection. Nevertheless, influenza vaccination uptake remains challenging in this population due to hesitancy and logistical barriers, particularly for those with reduced mobility or home confinement [54].

### 4.3 Hospitalization for COVID‐19 and Mortality Risk in Patients With Parkinson's Disease

Our analysis shows that Parkinson's disease patients hospitalized for COVID‐19 had a higher probability of in‐hospital death compared with other patients. Male sex and advanced age emerged as independent mortality risk factors, consistent with previous literature [55]. These findings underscore the particular vulnerability of Parkinson's disease patients to SARS‐CoV‐2 infection and highlight the need for targeted preventive measures, vigilant monitoring, and early management of complications.

Prior studies have reported increased COVID‐19 mortality among elderly and institutionalized Parkinson's disease patients. A Dutch study of long‐term care residents found an 18‐fold increased risk of death post‐SARS‐CoV‐2 infection, with Parkinson's disease, dementia, and male sex among the strongest mortality correlates; symptoms such as fever, dyspnea, delirium, and oxygen desaturation were also strongly associated with negative outcomes [56]. Fathi et al. [57] reported a 35.1% COVID‐19 mortality rate in Parkinson's disease patients versus 29.5% in non‐Parkinson's patients; decedents were older and more hypoxemic at admission. A German cross‐sectional study found 35.4% versus 20.7% mortality in Parkinson's versus non‐Parkinson's patients, with fatal Parkinson's cases being older, male, at more advanced disease stages, and having more comorbidities—particularly chronic kidney disease [58]. New York data showed 32% versus 26% mortality in Parkinson's versus non‐Parkinson's cohorts, with encephalopathy at admission more common in fatal Parkinson's cases [59]. An Iranian registry (*n* = 12 909; 87 Parkinson's cases) reported 35.6% mortality in Parkinson's versus 19.8% in others [60].

However, findings are not uniform. An Italian historical cohort reported an unadjusted OR for COVID‐19 mortality of 1.4 (95% CI = 0.6–3.0) in Parkinson's patients, which decreased to 0.9 (95% CI = 0.3–2.8) after adjustment, with confidence intervals crossing unity [46]. A retrospective cohort of 140 patients (12 with Parkinson's disease) found no fatalities among Parkinson's cases; most remained asymptomatic or mildly symptomatic [61]. El‐Qushayri et al. [62] meta‐analysis estimated a combined COVID‐19 mortality of 25.1% (95% CI = 16.37–38.49) in Parkinson's patients but no significant mortality difference versus non‐Parkinson's, suggesting comorbidities and age—rather than the neurological condition itself—drive risk.

One possible explanation for the higher COVID‐19 mortality observed in individuals with Parkinson's disease lies in the respiratory abnormalities associated with both conditions. Parkinson's disease can lead to respiratory impairments affecting both the airways and respiratory musculature, resulting in upper airway obstruction, obstructive sleep apnea, restrictive respiratory dysfunction, and aspiration pneumonia [63]. A systematic review of respiratory disorders in Parkinson's disease found that respiratory muscle weakness is present from the early stages of the disease and worsens as it progresses. Moreover, patients with Parkinson's disease are more likely to develop pulmonary dysfunction of both obstructive and restrictive types [63].

In the present study, patients with a diagnosis of Parkinson's disease had a higher prevalence of preexisting chronic respiratory diseases compared with other patients and were more prone to oxygen desaturation. These individuals also demonstrated a greater need for invasive mechanical ventilation support, which supports the hypothesis that clinically significant respiratory alterations are present in this group and may contribute to the poorer clinical outcomes observed.

### 4.4 Consequences of the COVID‐19 Pandemic for Patients With Parkinson's Disease: An Integrated Perspective

The COVID‐19 pandemic has profoundly affected the lives of individuals diagnosed with Parkinson's disease, primarily due to social‐isolation measures that led to the cancellation of medical appointments, physical‐therapy sessions, and social interactions. Although alternatives such as telemedicine and other digital tools were adopted, these resources proved less accessible to lower‐income and older patients [64].

A systematic review documented increased anxiety levels among Parkinson's disease patients during the pandemic, and other studies also reported a higher prevalence of depression, apathy, and sleep disturbances during this period [65]. A retrospective analysis of patients in Zurich demonstrated worsening of motor symptoms during the pandemic compared with the pre‐pandemic period [66].

Furthermore, a scoping review found that isolation‐related changes contributed to the deterioration of both motor and non‐motor symptoms, as well as the emergence of new clinical manifestations in Parkinson's disease patients. Factors associated with this decline included interruption of therapeutic and social activities, fear of infection (personal or among close contacts), and difficulty accessing reliable information and healthcare services. The pandemic's impact also extended to caregivers, who reported elevated anxiety and stress—especially those caring for individuals with more complex needs due to disease severity and cognitive or psychological changes [64].

According to a remote survey, 100% of Parkinson's disease patients who contracted COVID‐19 reported engaging in fewer recreational or work activities that might expose them to infection compared with individuals without the disease. Moreover, these patients were more likely to have household or other close contacts with confirmed, suspected, or symptomatic COVID‐19 cases [64]. The majority of their caregivers are women over 60 years of age [67, 68, 69]. Given that the pandemic can exacerbate both motor and non‐motor symptoms of Parkinson's disease, it is essential to provide support to both patients and caregivers, who often face substantial physical and emotional burdens.

Fereshtehnejad et al. [70] propose that telemedicine be used not only for medication management and acute symptom care but also as a platform for lifestyle counseling, well‐being discussions, support groups, rehabilitation services, and other complementary interventions. This approach broadens the scope of care and has been well received by Parkinson's disease populations even after the pandemic. However, challenges remain—particularly among older and socioeconomically disadvantaged patients—which can limit access to and effectiveness of these resources. Beyond telemedicine, wearable devices have gained prominence for diagnosis, assessment, and monitoring of Parkinson's disease patients. Such devices hold potential to enhance clinical follow‐up and should be more fully integrated by multidisciplinary care teams, contributing to a more comprehensive and personalized approach [71].

Vaccination is a cornerstone of protection against COVID‐19. Multiple studies show that SARS‐CoV‐2 vaccination is associated with reduced mortality, shorter hospital stays, and decreased need for intensive‐care support [72, 73, 74, 75]. Although some Parkinson's disease patients report transient worsening of motor symptoms after COVID‐19 vaccination, these effects are typically mild, self‐limited, and last only a few days. Such transient exacerbations have contributed to vaccine hesitancy and are often related to nonspecific immunization side effects [76]. The importance of COVID‐19 vaccination extends to other populations, such as residents of quilombos in Brazil, who have shown lower vaccination rates compared with the general population [77]. Moreover, concerns increase when considering the decline in global vaccination coverage observed in recent years, underscoring the need to promote broader awareness of the importance of mass vaccination for the control of infectious diseases [78].

An online survey in Shanghai found a 54% COVID‐19 vaccination rate among Parkinson's disease patients. Trust in government and concern for protecting others were the main drivers of acceptance, while fear of Parkinson's disease worsening and other comorbidities were the primary reasons for hesitancy [79]. In Brazil, a study examining political ideology and vaccine uptake found that support for a far‐right presidential candidate in 2018 and 2022 was inversely associated with COVID‐19 vaccination rates, illustrating how political beliefs, scientific mistrust, and misinformation can undermine national immunization efforts [80, 81].

Survivors of COVID‐19 with Parkinson's disease require longitudinal follow‐up, as they face higher risks of mortality, cardiovascular events, dyspnea, fatigue, and falls [82]. Infection may also worsen non‐motor Parkinson's symptoms—such as depression, anxiety, cognitive decline, and sleep disorders—highlighting the need for continuous, holistic care [83].

Although the database used in this study (Open‐Data‐SUS) has supported previous research and proven valuable [33, 35, 84, 85, 86, 87, 88, 89, 90, 91, 92, 93], further studies are needed to deepen our understanding of the COVID‐19–Parkinson's disease nexus. International collaboration—modeled by the United Kingdom National Institute for Health Research's rapid‐evidence approach during the pandemic—can expedite high‐impact research [94, 95, 96, 97, 98]. In addition, research should expand to other emerging pathogens in Brazil—such as influenza, monkeypox, *Bordetella pertussis*, Oropouche virus, and dengue virus—to assess their effects on vulnerable populations, including those with neurodegenerative conditions [99, 100, 101, 102].

### 4.5 Limitations

This retrospective observational study relies on Open‐Data‐SUS administrative records, which—while comprehensive—are subject to heterogeneous data entry, reporting bias, and under‐notification. The authors did not participate directly in data collection, limiting control over data quality and completeness. Only patients hospitalized with severe acute respiratory syndrome were included, excluding mild or asymptomatic cases; therefore, findings cannot be generalized to less severe COVID‐19 presentations. Although all Brazilian regions are represented, results may not apply to populations with differing socioeconomic contexts, epidemiological profiles, and healthcare systems. Vaccination status against COVID‐19 was included as a study variable. Information on whether each participant had received at least one dose of a COVID‐19 vaccine was available; however, data regarding the specific type of vaccine and the number of doses administered were not provided by the database.

Parkinson's disease diagnosis was self‐ or proxy‐reported at hospital admission without standardized verification, potentially underestimating prevalence—especially in regions with limited access to specialized neurology services. Important variables—such as disease duration, medication regimens, motor and non‐motor symptom profiles, socioeconomic status, education, and caregiver support—were unavailable but could significantly influence prognosis and analysis depth. Similarly, we do not have detailed information regarding other health conditions, nor about the specific treatments and medications used for each clinical comorbidity included in the study.

Data on antibacterial treatment were not available for the study population. We recognize that viral and bacterial co‐infections may have a combined effect on Parkinson's disease severity. The lack of information on antibacterial use is therefore acknowledged as a study limitation.

External factors over the 4‐year pandemic period—emergence of new SARS‐CoV‐2 variants, evolving vaccination coverage, advances in clinical management, and changing public‐health policies—may also have affected outcomes.

## 5 Conclusion

This study demonstrates that SARS‐CoV‐2 infection exerts a disproportionately adverse impact on individuals diagnosed with Parkinson's disease, particularly those hospitalized for severe COVID‐19. These patients exhibit higher rates of intensive‐care admission, greater need for invasive ventilatory support, and significantly elevated mortality. Male sex, advanced age, and heart disease (cardiopathy) and respiratory comorbidities were consistently associated with worse outcomes, underscoring the importance of enhanced surveillance and tailored management in this vulnerable population.

Given the multifactorial vulnerability of Parkinson's disease patients, integrated care strategies are imperative—encompassing continuous multidisciplinary follow‐up, maintenance of pharmacological and nonpharmacological interventions, and preservation of therapeutic and social routines, even amid pandemic constraints. Interruptions in these supports not only risk functional decline but may also exacerbate disease progression.

Moreover, it is urgent to strengthen public‐health policies to support individuals with Parkinson's disease—especially those in advanced stages—and their often overburdened caregivers. Investments in infrastructure, equitable access to specialized services, psychosocial support, and assistive technologies are essential to mitigate the impact of COVID‐19 and future health crises on this already fragile population.

## Author Contributions

L.S.M. and F.A.L.M. were responsible for data collection and tabulation. A.E.F.S., P.T.C., L.S.M., L.F.A.M., and F.A.L.M. contributed to the interpretation of the study findings. A.E.F.S., P.T.C., L.S.M., L.F.A.M., and F.A.L.M. were involved in drafting and critically revising the manuscript before submission. All authors approved the final version of the manuscript and consented to its submission to the scientific journal.

## Ethics Statement

The study was conducted in accordance with the principles of the Declaration of Helsinki and was approved by the Institutional Ethics Committee (Ethical approval number: 67241323.0.0000.5514; Study approval number: 5.908.611).

## Conflicts of Interest

The authors declare no conflicts of interest.

## Supporting information


**Supplementary Table 1:** Places of residence, case notification, and hospitalization of individuals hospitalized due to severe acute respiratory syndrome coronavirus 2 (SARS‐CoV‐2) infection in Brazil. **Supplementary Table 2:** Demographic characteristics, clinical signs and symptoms, comorbidities, and hospitalization data of individuals hospitalized due to severe acute respiratory syndrome coronavirus 2 (SARS‐CoV‐2) infection in Brazil, according to the presence of Parkinson's disease. **Supplementary Table 3:** Association between the time periods related to symptoms onset, hospitalization, clinical outcome, and length of stay in the intensive care unit with the diagnosis of Parkinson's disease and patient outcomes among individuals hospitalized due to severe acute respiratory syndrome coronavirus 2 (SARS‐CoV‐2) infection during the 4‐year coronavirus disease (COVID)‐19 pandemic period in Brazil. **Supplementary Table 4:** Multivariable analysis to determine the main predictors of Parkinson's disease diagnosis among those who were hospitalized due to severe acute respiratory syndrome coronavirus 2 (SARS‐CoV‐2) infection in Brazil during the 4‐year period of the pandemic. **Supplementary Table 5:** Demographic, clinical signs and symptoms, comorbidities, and hospitalization information of individuals hospitalized due to severe acute respiratory syndrome coronavirus 2 (SARS‐CoV‐2) infection in Brazil who evolve for death or hospital discharge (clinical recovery). **Supplementary Table 6:** yndrome coronavirus 2 (SARS‐CoV‐2) infection in Brazil during the 4‐year period of the pandemic.

## Data Availability

The materials used in this study are available from the corresponding author upon reasonable request.
