# Supplementary material for: Characterization of the Epidemiological Profile of Patients With Parkinson's Disease Who Were Hospitalized due to SARS‐CoV‐2 Infection: A Portrait of 4 Years of the Pandemic in Brazil
Source: J Med Virol. 2025 Nov 3;97(11):e70670. doi: 10.1002/jmv.70670 (PMC12581207; doi:10.1002/jmv.70670)
Supplement: Supplementary file 1 — Supplementary Table 1: Places of residence, case notification, and hospitalization of individuals hospitalized due to severe acute respiratory syndrome coronavirus 2 (SARS‐CoV‐2) infection in Brazil. Supplementary Table 2: Demographic characteristics, clinical signs and symptoms, comorbidities, and hospitalization data of individuals hospitalized due to severe acute respiratory syndrome coronavirus 2 (SARS‐CoV‐2) infection in Brazil, according to the presence of Parkinson's disease. Supplementary Table 3: Association between the time periods related to symptoms onset, hospitalization, clinical outcome, and length of stay in the intensive care unit with the diagnosis of Parkinson's disease and patient outcomes among individuals hospitalized due to severe acute respiratory syndrome coronavirus 2 (SARS‐CoV‐2) infection during the 4‐year coronavirus disease (COVID)‐19 pandemic period in Brazil. Supplementary Table 4: Multivariable analysis to determine the main predictors of Parkinson's disease diagnosis among those who were hospitalized due to severe acute respiratory syndrome coronavirus 2 (SARS‐CoV‐2) infection in Brazil during the 4‐year period of the pandemic. Supplementary Table 5: Demographic, clinical signs and symptoms, comorbidities, and hospitalization information of individuals hospitalized due to severe acute respiratory syndrome coronavirus 2 (SARS‐CoV‐2) infection in Brazil who evolve for death or hospital discharge (clinical recovery). Supplementary Table 6: yndrome coronavirus 2 (SARS‐CoV‐2) infection in Brazil during the 4‐year period of the pandemic. [file JMV-97-e70670-s001.docx]

**Supplementary Material**

**Title:** Characterization of the Epidemiological Profile of Patients with Parkinson’s Disease Who Were Hospitalized due to SARS-CoV-2 Infection: A Portrait of 4 of the Pandemic in Brazil

**Short title:** Parkinson’s disease and COVID-19

Adriele Evelyn Ferreira Silva ^1,2,3,#^; Patrícia Teixeira Costa ^1,2,3,#^; Lucas Silva Mello ^1,2,3,#^; Luiz Felipe Azevedo Marques ^1,2,3,#^; Fernando Augusto Lima Marson ^1,2,3,*,#^

^1^ Laboratory of Molecular Biology and Genetics, Postgraduate Program of Health Sciences, Postgraduate Program in Health Data Science, São Francisco University, Bragança Paulista, São Paulo, Brazil.

^2^ Laboratory of Clinical and Molecular Microbiology, Postgraduate Program of Health Sciences, Postgraduate Program in Health Data Science, São Francisco University, Bragança Paulista, São Paulo, Brazil.

^3^ LunGuardian Research Group — Epidemiology of Respiratory and Infectious Diseases, Postgraduate Program of Health Sciences, Postgraduate Program in Health Data Science, São Francisco University, Bragança Paulista, São Paulo, Brazil.

*** Corresponding Author:** [FALM] Fernando Augusto Lima Marson, BSc, MSc, PhD.

Laboratory of Molecular Biology and Genetics, Laboratory of Clinical and Molecular Microbiology, LunGuardian Research Group — Epidemiology of Respiratory and Infectious Diseases, Postgraduate Program of Health Sciences, Postgraduate Program in Health Data Science, São Francisco University. Avenida São Francisco de Assis, 218. Jardim São José, Bragança Paulista 12916-900, São Paulo, Brasil. Phone number: +55-19-999752911.

**E-mail:** fernandolimamarson@hotmail.com and fernando.marson@usf.edu.br.

^#^ The authors contributed equally to this study.

**Study authors’ e-mails and ORCIDs:**

**AEFS:** adriele.da@mail.usf.edu.br

**ORCID:** 0000-0003-1215-6427

**PTC:** patricia.costa@usf.edu.br

ORCID: 0009-0002-8179-5644

**LSM:** lucas.silva.mello@mail.usf.edu.br

ORCID: 0009-0006-9920-5058

**LFAM:** luiz.azevedo@mail.usf.edu.br

ORCID: 0009-0008-5494-2171

**FALM**: fernandolimamarson@hotmail.com and fernando.marson@usf.edu.br

ORCID: 0000-0003-4955-4234

| **Supplementary Table 1.** Places of residence, case notification, and hospitalization of individuals hospitalized due to severe acute respiratory syndrome coronavirus 2 (SARS-CoV-2) infection in Brazil. | | | |
| --- | --- | --- | --- |
| **Federative units** | **Place of residence** | **Place of notification** | **Place of hospitalization** |
| **Central-West region** | **171,969 (10.1%)** | **171,994 (9.9%)** | **169,803 (9.8%)** |
| Federal District | 38,963 (2.3%) | 41,906 (2.4%) | 41,239 (2.4%) |
| Goiás | 71,726 (4.2%) | 69,567 (4.0%) | 68,776 (4.0%) |
| Mato Grosso | 30,884 (1.8%) | 30,437 (1.8%) | 29,892 (1.7%) |
| Mato Grosso do Sul | 30,396 (1.8%) | 30,084 (1.7%) | 29,896 (1.7%) |
| **North region** | **107,736 (6.3%)** | **106,033 (6.2%)** | **104,529 (6.0%)** |
| Acre | 4297 (0.2%) | 4343 (0.3%) | 4279 (0.2%) |
| Amapá | 4408 (0.3%) | 4537 (0.3%) | 4506 (0.3%) |
| Amazonas | 32,204 (1.9%) | 31,420 (1.8%) | 31,069 (1.8%) |
| Pará | 41,791 (2.4%) | 41,040 (2.4%) | 40,512 (2.3%) |
| Rondônia | 12,993 (0.8%) | 12,692 (0.7%) | 12,525 (0.7%) |
| Roraima | 3592 (0.2%) | 3549 (0.2%) | 3544 (0.2%) |
| Tocantins | 8451 (0.5%) | 8452 (0.5%) | 8094 (0.5%) |
| **Northest region** | **268,533 (15.6%)** | **268,306 (15.6%)** | **263,038 (15.3%)** |
| Alagoas | 17,496 (1.0%) | 17,389 (1.0%) | 17,129 (1.0%) |
| Bahia | 62,445 (3.6%) | 62,272 (3.6%) | 61,076 (3.5%) |
| Ceará | 58,979 (3.4%) | 59,034 (3.4%) | 56,958 (3.3%) |
| Maranhão | 19,221 (1.1%) | 18,657 (1.1%) | 18,419 (1.1%) |
| Paraíba | 23,577 (1.4%) | 23,590 (1.4%) | 23,323 (1.4%) |
| Pernambuco | 42,563 (2.5%) | 42,529 (2.5%) | 41,691 (2.4%) |
| Piauí | 17,077 (1.0%) | 17,556 (1.0%) | 17,412 (1.0%) |
| Rio Grande do Norte | 16,621 (1.0%) | 16,621 (1.0%) | 16,513 (1.0%) |
| Sergipe | 10,554 (0.6%) | 10,658 (0.6%) | 10,517 (0.6%) |
| **Southest region** | **877,116 (50.9%)** | **879,296 (51.0%)** | **856,023 (50.1%)** |
| Espírito Santo | 13,226 (0.8%) | 13,131 (0.8%) | 12,718 (0.7%) |
| Minas Gerais | 188,405 (10.9%) | 187,716 (10.9%) | 185,739 (10.8%) |
| Rio de Janeiro | 156,753 (9.1%) | 156,529 (9.1%) | 153,839 (8.9%) |
| São Paulo | 518,732 (30.1%) | 521,920 (30.2%) | 512,727 (29.7%) |
| **South region** | **300,092 (17.4%)** | **300,061 (17.4%)** | **297,067 (17.2%)** |
| Paraná | 117,535 (6.8%) | 117,539 (6.8%) | 116,914 (6.8%) |
| Rio Grande do Sul | 112,361 (6.5%) | 112,181 (6.5%) | 110,758 (6.4%) |
| Santa Catarina | 70,196 (4.1%) | 70,341 (4.1%) | 69,395 (4.0%) |
| **Unknown (not informed)** | 244 (0.0%) | None | 26,230 (1.5%) |
| **Total** | 1,725,690 (100%) | 1,725,690 | 1,725,690 (100%) |

Data are presented as absolute frequencies (N) and relative frequencies (%, percentages).

The epidemiological analysis was based on data from Open-Data-SUS (https://opendatasus.saude.gov.br/), covering a 4-year period of the coronavirus disease (COVID)-19 pandemic in Brazil (from February 22, 2020, to May 24, 2024).

| **Supplementary Table 2.** Demographic characteristics, clinical signs and symptoms, comorbidities, and hospitalization data of individuals hospitalized due to severe acute respiratory syndrome coronavirus 2 (SARS-CoV-2) infection in Brazil, according to the presence of Parkinson’s disease. | | | | | | |
| --- | --- | --- | --- | --- | --- | --- |
| **Markers** | **Categories** | **Patients with Parkinson**’s **disease** | **Other patients** | **Total** | **P-value** | **OR (95% CI)** |
| Parkinson’s disease | Yes | 4907 (0.3%) | 1,720,783 (99.7%) | 1,725,690 | — | — |
| Vaccination against coronavirus disease (COVID)-19 | Yes | 2152 (43.9%) | 399,788 (23.2%) | 401,940 (23.3%) | **<0.001** | **2.581 (2.439 to 2.731)** |
|  | No | 2755 (56.1%) | 1,320,995 (76.8%) | 1,323,750 (76.7%) |  | 1 (reference) |
| Sociodemographic | | |  |  |  |  |
| Sex | Male | 2828 (57.6%) | 942,917 (54.8%) | 945,745 (54.8%) | **<0.001** | **1.122 (1.060 to 1.188)** |
|  | Female | 2079 (42.4%) | 777,866 (45.2%) | 779,945 (42.2%) |  | 1 (reference) |
| Age | 40 to 59 years of age | 192 (3.9%) | 693,445 (40.3%) | 693,637 (40.2%) |  | 1 (reference) |
|  | 60 to 64 years of age | 222 (4.5%) | 197,559 (11.5%) | 197,781 (11.5%) | **<0.001** | **4.059 (3.345. to 4.924)** |
|  | 65 to 69 years of age | 423 (8.6%) | 198,375 (11.5%) | 198,798 (11.5%) | **<0.001** | **7.701 (6.493 to 9.134)** |
|  | 70 to 74 years of age | 680 (13.9%) | 183,267 (10.7%) | 183,947 (10.7%) | **<0.001** | **13.401 (11.417 to 15.730)** |
|  | 75 to 79 years of age | 956 (19.5%) | 155,200 (9.0%) | 156,156 (9.0%) | **<0.001** | **22.247 (19.041 to 25.980)** |
|  | 80 to 84 years of age | 1095 (22.3%) | 130,298 (7.6%) | 131,393 (7.6%) | **<0.001** | **30.352 (26.034 to 35.386)** |
|  | 85 to 90 years of age | 833 (17.0%) | 93,249 (5.4%) | 94,082 (5.5%) | **<0.001** | **32.264 (27.574 to 37.750)** |
|  | +90 years of age | 506 (10.3%) | 69,390 (4.0%) | 69,896 (4.1%) | **<0.001** | **26.337 (22.302 to 31.102)** |
| Race | White people | 3412 (69.5%) | 1,030,262 (59.9%) | 1,033,674 (59.9%) |  | 1 (reference) |
|  | Black people | 107 (2.2%) | 74,064 (4.3%) | 74,171 (4.3%) | **<0.001** | **0.436 (0.360 to 0.529)** |
|  | Asian individuals | 52 (1.1%) | 17,202 (1.0%) | 17,254 (1.0%) | 0.514 | 0.913 (0.694 to 1.201) |
|  | Mixed individuals^*^ | 1330 (27.1%) | 596,366 (34.7%) | 597,696 (34.6%) | **<0.001** | **0.673 (0.632 to 0.718)** |
|  | Indigenous peoples | 6 (0.1%) | 2889 (0.2%) | 2895 (0.2%) | 0.254 | 0.627 (0.281 to 1.398) |
| Geographic zone | Urban | 4713 (96.0%) | 1,639,415 (95.3%) | 1,644,128 (95.3%) | **0.011** | **1.206 (1.044 to 1.392)** |
|  | Rural + peri-urban | 194 (4.0%) | 81,368 (4.7%) | 81,562 (4.7%) |  | 1 (reference) |
| Nosocomial infection | Yes | 111 (2.3%) | 35,011 (2.0%) | 35,122 (2.0%) | 0.260 | 1.114 (0.923 to 1.346) |
|  | No | 4796 (97.7%) | 1,685,772 (98.0%) | 1,690,568 (98.0%) |  | 1 (reference) |
| Clinical signs and symptoms |  |  |  |  |  |  |
|  | Fever | 3443 (70.2%) | 1,230,299 (71.5%) | 1,233,742 (71.5%) | **0.039** | **0.938 (0.882 to 0.997)** |
|  | Cough | 3774 (76.9%) | 1,402,527 (81.5%) | 1,406,301 (81.5%) | **<0.001** | **0.756 (0.707 to 0.808)** |
|  | Sore throat | 480 (9.8%) | 284,834 (16.6%) | 285,314 (16.5%) | **<0.001** | **0.547 (0.497 to 0.601)** |
|  | Dyspnea | 3896 (79.4%) | 1,431,857 (83.2%) | 1,435,753 (83.2%) | **<0.001** | **0.778 (0.726 to 0.833)** |
|  | Respiratory discomfort | 3618 (73.7%) | 1,299,914 (75.5%) | 1,303,532 (75.5%) | **0.003** | **0.909 (0.853 to 0.969)** |
|  | Oxygen saturation < 95% | 4017 (81.9%) | 1,383,599 (80.4%) | 1,387,616 (80.4%) | **0.010** | **1.100 (1.023 to 1.183)** |
|  | Diarrhea | 421 (8.6%) | 210,936 (12.3%) | 211,357 (12.2%) | **<0.001** | **0.672 (0.608 to 0.742)** |
|  | Vomiting | 281 (5.7%) | 128,716 (7.5%) | 128,997 (7.5%) | **<0.001** | **0.751 (0.666 to 0.848)** |
|  | Fatigue | 858 (17.5%) | 382,457 (22.2%) | 383,315 (22.2%) | **<0.001** | **0.742 (0.689 to 0.798)** |
|  | Other symptoms | 1960 (39.9%) | 550,193 (32.0%) | 552,153 (32.0%) | **<0.001** | **1.415 (1.336 to 1.498)** |
| Comorbidities |  |  |  |  |  |  |
|  | Heart disease (cardiopathy) | 1859 (37.9%) | 627,299 (36.5%) | 629,158 (36.5%) | **0.038** | **1.063 (1.003 to 1.126)** |
|  | Hematological disorder | 33 (0.7%) | 11,682 (0.7%) | 11,715 (0.7%) | 0.957 | 0.991 (0.703 to 1.396) |
|  | Down syndrome | 6 (0.1%) | 3,501 (0.2%) | 3,507 (0.2%) | 0.212 | 0.601 (0.269 to 1.338) |
|  | Hepatic disorder | 32 (0.7%) | 14,533 (0.8%) | 14,565 (0.8%) | 0.142 | 0.771 (0.544 to 1.091) |
|  | Asthma | 84 (1.7%) | 38,333 (2.2%) | 38,417 (2.2%) | **0.015** | **0.764 (0.616 to 0.949)** |
|  | Diabetes mellitus | 1217 (24.8%) | 442,292 (25.7%) | 443,509 (25.7%) | 0.149 | 0.953 (0.893 to 1.017) |
|  | Chronic respiratory disease | 220 (4.5%) | 66,434 (3.9%) | 66,654 (3.9%) | **0.024** | **1.169 (1.021 to 1.338)** |
|  | Immunosuppression disorder | 102 (2.1%) | 41,408 (2.4%) | 41,510 (2.4%) | 0.135 | 0.861 (0.707 to 1.048) |
|  | Kidney disease | 164 (3.3%) | 66,631 (3.9%) | 66,795 (3.9%) | 0.055 | 0.858 (0.735 to 1.003) |
|  | Obesity | 141 (2.9%) | 128,045 (7.4%) | 128,186 (7.4%) | **<0.001** | **0.368 (0.311 to 0.435)** |
| Received antiviral medication for the Flu | Yes | 285 (5.8%) | 111,298 (6.5%) | 111,583 (6.5%) | 0.061 | 0.892 (0.791 to 1.005) |
|  | No | 4622 (94.2%) | 1,609,485 (93.5%) | 1,614,107 (93.5%) |  | 1 (reference) |
| Need for intensive care unit | Yes | 1934 (39.4%) | 590,025 (34.3%) | 591,959 (34.3%) | **<0.001** | **1.247 (1.177 to 1.320)** |
|  | No | 2973 (60.6%) | 1,130,758 (65.7%) | 1,133,731 (65.7%) |  | 1 (reference) |
| Need to mechanical ventilatory support | Invasive | 992 (20.2%) | 325,917 (18.9%) | 326,963 (18.9%) | **0.023** | **1.115 (1.015 to 1.224)** |
|  | Noninvasive | 3122 (63.6%) | 1,104,375 (64.2%) | 1,107,497 (64.2%) | 0.383 | 1.035 (0.958 to 1.119) |
|  | No | 793 (16.2%) | 290,437 (16.9%) | 291,230 (16.9%) |  | 1 (reference) |
| Outcome | Death | 2607 (53.1%) | 638,750 (37.1%) | 641,357 (37.2%) | **<0.001** | **1.920 (1.815 to 2.031)** |
|  | Hospital discharge | 2300 (46.9%) | 1,082,033 (62.9%) | 1,084,333 (62.8%) |  | 1 (reference) |

*: Individuals with a multiracial background (*Pardos*).

Data are presented as absolute frequencies (N) and relative frequencies (%, percentage).

The association between markers was assessed using the Odds Ratio (OR) and the 95% confidence interval (95% CI). Statistical analysis was performed using logistic regression. A p-value of 0.05 was considered statistically significant. Statistically significant results are highlighted in bold in the table for ease of interpretation.

The epidemiological analysis was based on data from Open-Data-SUS (https://opendatasus.saude.gov.br/), covering a 4-year period of the COVID-19 pandemic in Brazil (from February 22, 2020, to May 24, 2024).

| **Supplementary Table 3.** Association between the time periods related to symptoms onset, hospitalization, clinical outcome, and length of stay in the intensive care unit with the diagnosis of Parkinson’s disease and patient outcomes among individuals hospitalized due to severe acute respiratory syndrome coronavirus 2 (SARS-CoV-2) infection during the 4-year coronavirus disease (COVID)-19 pandemic period in Brazil. | | | |
| --- | --- | --- | --- |
| **Period of time between …** | **Patients with Parkinson**’**s disease** | **Other patients** | **P-value** |
| … date of admission and date of onset of symptoms | 5.89 (5.67 to 6.12) | 9.95 (7.40 to 12.51) | 0.868 |
| … date of outcome and date of onset of symptoms | 18.26 (17.76 to 18.77) | 19.22 (19.19 to 19.25) | **< 0.001** |
| … date of outcome and date of admission | 12.59 (12.14 to 13.03) | 12.11 (12.08 to 12.13) | 0.053 |
| … date of discharge and date of admission to the intensive care unit | 3.24 (3.04 to 3.45) | 3.27 (3.26 to 3.29) | 0.764 |
| **Period of time between …** | **Death** | **Hospital discharge** | **P-value** |
| … date of admission and date of onset of symptoms | 6.95 (6.92 to 6.97) | 11.71 (7.66 to 15.76) | **< 0.001** |
| … date of outcome and date of onset of symptoms | 19.43 (19.38 to 19.48) | 19.10 (19.06 to 19.13) | **< 0.001** |
| … date of outcome and date of admission | 13.12 (13.09 to 13.16) | 11.51 (11.48 to 11.54) | **< 0.001** |
| … date of discharge and date of admission to the intensive care unit | 5.20 (5.17 to 5.22) | 2.14 (2.13 to 2.15) | **< 0.001** |
| **Period of time between …** | **All individuals** | | **P-value** |
| … date of admission and date of onset of symptoms | 9.94 (7.34 to 12.49) | | NA |
| … date of outcome and date of onset of symptoms | 19.22 (19.19 to 19.24) | | NA |
| … date of outcome and date of admission | 12.11 (12.08 to 12.14 | | NA |
| … date of discharge and date of admission to the intensive care unit | 3.27 (3.26 to 3.28) | | NA |

N: not applicable.

Data are presented as mean values with the corresponding 95% confidence interval (95% CI). Statistical analysis was performed using Student’s T-test. A p-value of 0.05 was considered statistically significant. Statistically significant results are highlighted in bold in the table for ease of interpretation.

The epidemiological analysis was based on data from Open-Data-SUS (https://opendatasus.saude.gov.br/), covering a 4-year period of the coronavirus disease (COVID)-19 pandemic in Brazil (from February 22, 2020, to May 24, 2024).

| **Supplementary Table 4.** Multivariable analysis to determine the main predictors of Parkinson’s disease diagnosis among those who were hospitalized due to severe acute respiratory syndrome coronavirus 2 (SARS-CoV-2) infection in Brazil during the 4-year period of the pandemic.^a^ | | | | | | |
| --- | --- | --- | --- | --- | --- | --- |
| **Markers** | **β** | **SE** | **Wald** | **DF** | **P-value** | **OR (95% CI)** |
| Received vaccination against coronavirus disease (COVID)-19 | 0.531 | 0.030 | 315.031 | 1 | **< 0.001** | **1.701 (1.604 to 1.803)** |
| Sex (Male) | -0.277 | 0.029 | 89.475 | 1 | **< 0.001** | **0.758 (0.715 to 0.803)** |
| Age groups |  |  |  |  |  |  |
| 40 to 59 years of age |  |  | 2581.546 | 7 | **< 0.001** |  |
| 60 to 64 years of age | 1.384 | 0.099 | 196.373 | 1 | **< 0.001** | **3.993 (3.290 to 4.846)** |
| 65 to 69 years of age | 1.995 | 0.088 | 519.586 | 1 | **< 0.001** | **7.354 (6.195 to 8.730)** |
| 70 to 74 years of age | 2.514 | 0.083 | 927.021 | 1 | **< 0.001** | **12.360 (10.513 to 14.532)** |
| 75 to 79 years of age | 2.992 | 0.080 | 1388.073 | 1 | **< 0.001** | **19.922 (17.021 to 23.318)** |
| 80 to 84 years of age | 3.277 | 0.080 | 1684.507 | 1 | **< 0.001** | **26.492 (22.655 to 30.980)** |
| 85 to 90 years of age | 3.302 | 0.082 | 1620.051 | 1 | **< 0.001** | **27.170 (23.134 to 31.909)** |
| +90 years of age | 3.053 | 0.087 | 1224.418 | 1 | **< 0.001** | **21.183 (17.854 to 25.134)** |
| Race |  |  |  |  |  |  |
| White people |  |  | 112.638 | 4 | **< 0.001** |  |
| Black people | -0.701 | 0.098 | 50.744 | 1 | **< 0.001** | **0.496 (0.409 to 0.601)** |
| Asian individuals | -0.141 | 0.140 | 1.012 | 1 | 0.314 | 0.868 (0.660 to 1.143) |
| Mixed individuals^*^ | -0.279 | 0.033 | 72.085 | 1 | **< 0.001** | **0.756 (0.709 to 0.807)** |
| Indigenous peoples | -0.341 | 0.411 | 0.690 | 1 | 0.406 | 0.711 (0.318 to 1.590) |
| Place of residence (Urban) | 0.267 | 0.074 | 13.030 | 1 | **< 0.001** | **1.306 (1.130 to 1.509)** |
| Clinical signs and symptoms |  |  |  |  |  |  |
| Fever | 0.157 | 0.034 | 20.662 | 1 | **< 0.001** | **1.170 (1.093 to 1.251)** |
| Cough | -0.162 | 0.037 | 18.596 | 1 | **< 0.001** | **0.851 (0.790 to 0.916)** |
| Sore throat | -0.316 | 0.049 | 41.906 | 1 | **< 0.001** | **0.729 (0.662 to 0.802)** |
| Dyspnea | -0.258 | 0.040 | 40.603 | 1 | **< 0.001** | **0.773 (0.714 to 0.837)** |
| Peripheral oxygen saturation < 95% | 0.125 | 0.042 | 8.775 | 1 | **0.003** | **1.134 (1.043 to 1.232)** |
| Diarrhea | -0.144 | 0.053 | 7.468 | 1 | **0.006** | **0.866 (0.781 to 0.960)** |
| Vomiting | -0.112 | 0.063 | 3.138 | 1 | 0.076 | 0.894 (0.790 to 1.012) |
| Fatigue | -0.184 | 0.038 | 23.002 | 1 | **< 0.001** | **0.832 (0.772 to 0.897)** |
| Comorbidities |  |  |  |  |  |  |
| Heart disease (cardiopathy) | -0.318 | 0.030 | 112.369 | 1 | **< 0.001** | **0.727 (0.686 to 0.771)** |
| Chronic respiratory disease | -0.315 | 0.070 | 20.468 | 1 | **< 0.001** | **0.730 (0.637 to 0.837)** |
| kidney disease | -0.430 | 0.080 | 28.862 | 1 | **< 0.001** | **0.650 (0.556 to 0.761)** |
| Obesity | -0.441 | 0.086 | 26.025 | 1 | **< 0.001** | **0.643 (0.543 to 0.762)** |
| Need for intensive care unit admission | 0.152 | 0.034 | 20.028 | 1 | **< 0.001** | **1.164 (1.089 to 1.244)** |
| Needed ventilatory support |  |  |  |  |  |  |
| Invasive | -0.115 | 0.056 | 4.108 | 1 | **0.043** | **0.892 (0.798 to 0.996)** |
| Noninvasive | 0.009 | 0.042 | 0.042 | 1 | 0.838 | 0.992 (0.913 to 1.076) |
| None |  |  | 6.371 | 2 | **0.041** |  |
| Outcome | 0.244 | 0.032 | 57.054 | 1 | **< 0.001** | **1.277 (1.198 to 1.360)** |
| Constant | -8.048 | 0.114 | 4949.092 | 1 | < 0.001 |  |

*: Mixed refers to individuals with a multiracial background (*Pardos*). β: the average increase in the outcome per unit increase of the predictor; DF: degrees of freedom; SE: standard error.

The association between markers is described using the Odds Ratio (OR) and the 95% confidence interval (95% CI). Multivariable analysis was performed using the Binary Logistic Regression Model with the Backward Stepwise method. Markers with a p-value ≤ 0.05 in the bivariate analysis were included in the regression model. The response variable was the Parkinson’s disease diagnosis.

Data for clinical signs and symptoms (others) and patient characteristics with a p-value > 0.05 were excluded from the analysis. A p-value of. 0.05 was considered statistically significant. Statistically significant data are marked in bold in the table for ease of interpretation.

The epidemiological analysis was based on data from Open-Data-SUS (https://opendatasus.saude.gov.br/), covering a 4-year period of the COVID-19 pandemic in Brazil (from February 22, 2020, to May 24, 2024).

^a^, Markers included in step 1 of the multivariable analysis were: COVID-19 vaccination status, sex, age group, race, place of residence, clinical signs and symptoms (fever, cough, sore throat, dyspnea, respiratory discomfort, peripheral oxygen saturation < 95%, diarrhea, vomiting, and fatigue), comorbidities [heart disease (cardiopathy), asthma, chronic lung disease, and obesity], need for intensive care unit admission, use of ventilatory support, outcome, and time intervals between outcome and symptom onset.

| **Supplementary Table 5.** Demographic, clinical signs and symptoms, comorbidities, and hospitalization information of individuals hospitalized due to severe acute respiratory syndrome coronavirus 2 (SARS-CoV-2) infection in Brazil who evolve for death or hospital discharge (clinical recovery). | | | | | | |
| --- | --- | --- | --- | --- | --- | --- |
| **Markers** | **Categories** | **Death** | **Hospital discharge** | **Total** | **P-value** | **OR (95% CI)** |
| Parkinson’s disease | Yes | 2607 (0.4%) | 2300 (0.7%) | 4907 (0.3%) | **<0.001** | **1.920 (1.815 to 2.031)** |
|  | No | 638,750 (99.6%) | 1,082,033 (99.3%) | 1,720,783 (99.7%) |  | 1 (reference) |
| Vaccination against coronavirus disease (COVID)-19 | Yes | 134,939 (21.0%) | 267,001 (24.6%) | 401,940 (23.3%) | **<0.001** | **0.816 (0.810 to 0.822)** |
|  | No | 506,418 (79.0%) | 817,332 (75.4%) | 1,323,750 (76.7%) |  | 1 (reference) |
| Sociodemographic | | |  |  |  |  |
| Sex | Male | 357,135 (55.7%) | 588,610 (54.3%) | 945,745 (54.8%) | **<0.001** | **1.058 (1.052 to 1.065)** |
|  | Female | 284,222 (44.3%) | 495,723 (45.7%) | 779,945 (45.2%) |  | 1 (reference) |
| Age | 40 to 59 years of age | 165,529 (25.8%) | 528,108 (48.7%) | 693,637 (40.2%) |  | 1 (reference) |
|  | 60 to 64 years of age | 70,757 (11.0%) | 127,024 (11.7%) | 197,781 (11.5%) | **<0.001** | **1.777 (1.758 to 1.796)** |
|  | 65 to 69 years of age | 82,105 (12.8%) | 116,693 (10.8%) | 198,798 (11.5%) | **<0.001** | **2.245 (2.221 to 2.268)** |
|  | 70 to 74 years of age | 83,972 (13.1%) | 99,975 (9.2%) | 183,947 (10.7%) | **<0.001** | **2.680 (2.651 to 2.709)** |
|  | 75 to 79 years of age | 76,832 (12.0%) | 79,324 (7.3%) | 156,156 (9.0%) | **<0.001** | **3.090 (3.055 to 3.125)** |
|  | 80 to 84 years of age | 68,766 (10.7%) | 62,627 (5.8%) | 131,393 (7.6%) | **<0.001** | **3.503 (3.461 to 3.546)** |
|  | 85 to 90 years of age | 51,801 (8.1%) | 42,281 (3.9%) | 94,082 (5.5%) | **<0.001** | **3.909 (3.855 to 3.964)** |
|  | +90 years of age | 41,595 (6.5%) | 28,301 (2.6%) | 69,896 (4.1%) | **<0.001** | **4.689 (4.614 to 4.765)** |
| Race | White people | 364,357 (56.8%) | 669,317 (61.7%) | 1,033,674 (59.9%) |  | 1 (reference) |
|  | Black people | 32,330 (5.0%) | 41,841 (3.9%) | 74,171 (4.3%) | **<0.001** | **1.419 (1.398 to 1.441)** |
|  | Asian individuals | 6183 (1.0%) | 11,071 (1.0%) | 17,254 (1.0%) | 0.110 | 1.026 (0.994 to 1.059) |
|  | Mixed individuals^*^ | 237,226 (37.0%) | 360,470 (33.2%) | 597,696 (34.6%) | **<0.001** | **1.209 (1.201 to 1.217)** |
|  | Indigenous peoples | 1261 (0.2%) | 1634 (0.2%) | 2895 (0.2%) | **<0.001** | **1.418 (1.317 to 1.526)** |
| Geographic zone | Urban | 608,709 (94.9%) | 1,035,419 (95.5%) | 1,644,128 (95.3%) | **<0.001** | **0.881 (0.868 to 0.894)** |
|  | Rural + peri-urban | 32,648 (5.1%) | 48,914 (4.5%) | 81,562 (4.7%) |  | 1 (reference) |
| Nosocomial infection | Yes | 16,612 (2.6%) | 18,510 (1.7%) | 35,122 (2.0%) | **<0.001** | **1.531 (1.499 to 1.564)** |
|  | No | 624,745 (97.4%) | 1,065,823 (98.3%) | 1,690,568 (98.0%) |  | 1 (reference) |
| Clinical signs and symptoms |  |  |  |  |  |  |
|  | Fever | 455,508 (71.0%) | 778,234 (71.8%) | 1,233,472 (71.5%) | **<0.001** | **0.964 (0.957 to 0.971)** |
|  | Cough | 510,760 (79.6%) | 895,541 (82.6%) | 1,406,301 (81.5%) | **<0.001** | **0.824 (0.818 to 0.831)** |
|  | Sore throat | 91,239 (14.2%) | 194,075 (17.9%) | 285,314 (16.6%) | **<0.001** | **0.761 (0.754 to 0.767)** |
|  | Dyspnea | 564,240 (88.0%) | 871,513 (80.4%) | 1,435,753 (83.2%) | **<0.001** | **1.787 (1.771 to 1.803)** |
|  | Respiratory discomfort | 522,940 (81.5%) | 780,592 (72.0%) | 1,303,532 (75.5%) | **<0.001** | **1.718 (1.705 to 1.731)** |
|  | Oxygen saturation < 95% | 552,547 (86.2%) | 835,069 (77.0%) | 1,387,616 (80.4%) | **<0.001** | **1.857 (1.842 to 1.873)** |
|  | Diarrhea | 67,644 (10.5%) | 143,713 (13.3%) | 211,357 (12.3%) | **<0.001** | **0.772 (0.764 to 0.779)** |
|  | Vomiting | 42,478 (6.6%) | 86,519 (8.0%) | 128,997 (7.5%) | **<0.001** | **0.818 (0.808. to 0.828)** |
|  | Fatigue | 132,602 (20.7%) | 250,713 (23.1%) | 383,315 (22.2%) | **<0.001** | **0.867 (0.860 to 0.873)** |
|  | Other symptoms | 179,010 (27.9%) | 373,143 (34.4%) | 552,153 (32.0%) | **<0.001** | **0.738 (0.733 to 0.743)** |
| **Comorbidities** |  |  |  |  |  |  |
|  | Heart disease (cardiopathy) | 268,755 (41.9%) | 360,403 (33.2%) | 629,158 (36.5%) | **<0.001** | **1.449 (1.440 to 1.458)** |
|  | Hematological disorder | 5560 (0.9%) | 6155 (0.6%) | 11,715 (0.7%) | **<0.001** | **1.532 (1.477 to 1.589)** |
|  | Down syndrome | 1528 (0.2%) | 1979 (0.2%) | 3507 (0.2%) | **<0.001** | **1.306 (1.222 to 1.396)** |
|  | Hepatic disorder | 7824 (1.2%) | 6741 (0.6%) | 14,565 (0.8%) | **<0.001** | **1.974 (1.911 to 2.040)** |
|  | Asthma | 12,786 (2.0%) | 25,631 (2.4%) | 38,417 (2.2%) | **<0.001** | **0.840 (0.822 to 0.858)** |
|  | Diabetes mellitus | 193,122 (30.1%) | 250,387 (23.1%) | 443,509 (25.7%) | **<0.001** | **1.435 (1.425 to 1.445)** |
|  | Chronic respiratory disease | 34,419 (5.4%) | 32,238 (3.0%) | 66,654 (3.9%) | **<0.001** | **1.851 (1.822 to 1.879)** |
|  | Immunosuppression disorder | 20,518 (3.2%) | 20,992 (1.9%) | 41,510 (2.4%) | **<0.001** | **1.674 (1.642 to 1.707)** |
|  | Kidney disease | 37,317 (5.8%) | 29,478 (2.7%) | 66,795 (3.9%) | **<0.001** | **2.211 (2.177 to 2.245)** |
|  | Obesity | 55,037 (8.6%) | 73,149 (6.7%) | 128,186 (7.4%) | **<0.001** | **1.298 (1.283 to 1.313)** |
| Received antiviral medication for the Flu | Yes | 40,783 (6.4%) | 70,800 (6.5%) | 111,583 (6.5%) | **<0.001** | **0.972 (0.960 to 0.984)** |
|  | No | 600,574 (93.6%) | 1,013,533 (93.5%) | 1,614,107 (93.5%) |  | 1 (reference) |
| Need for intensive care unit | Yes | 351,211 (54.8%) | 240,748 (22.2%) | 591,959 (34.3%) | **<0.001** | **4.241 (4.213 to 4.270)** |
|  | No | 290,146 (45,2%) | 843,585 (77.8%) | 1,133,731 (65.7%) |  | 1 (reference) |
| Need to mechanical ventilatory support | Invasive | 260,973 (40.7%) | 65,990 (6.1%) | 326,963 (18.9%) | **<0.001** | **19.657 (19.404 to 19.913)** |
|  | Noninvasive | 331,605 (51.7%) | 775,892 (71.6%) | 1,107,497 (64.2%) | **<0.001** | **2.124 (2.102 to 2.147)** |
|  | No | 48,779 (7.6%) | 242,451 (22.4%) | 291,230 (16.9%) |  | 1 (reference) |

*: Individuals with a multiracial background (*Pardos*).

Data are presented as absolute frequency (N) and relative frequency (%, percentage). The association between markers was assessed using the Odds Ratio (OR) and the 95% confidence interval (95% CI). The statistical analysis was performed using logistic regression. A p-value of 0.05 was considered statistically significant. Statistically significant results are highlighted in bold in the table for ease of interpretation.

The epidemiological analysis was conducted using data from Open-Data-SUS (https://opendatasus.saude.gov.br/), covering a 4-year period of the COVID-19 pandemic in Brazil (from February 22, 2020, to May 24, 2024).

| **Supplementary Table 6.** Multivariable analysis to determine the main predictors of death among those who were hospitalized due to severe acute respiratory syndrome coronavirus 2 (SARS-CoV-2) infection in Brazil during the 4-year period of the pandemic.^a^ | | | | | | |
| --- | --- | --- | --- | --- | --- | --- |
| **Markers** | **β** | **SE** | **Wald** | **DF** | **P-value** | **OR (95% CI)** |
| Parkinson’ disease | 0.268 | 0.033 | 67.506 | 1 | **< 0.001** | **1.307 (1.226 to 1.393)** |
| Received vaccination against coronavirus disease (COVID)-19 | -0.555 | 0.005 | 14,081.191 | 1 | **< 0.001** | **0.574 (0.569 to 0.579)** |
| Sex (male) | 0.148 | 0.004 | 1517.099 | 1 | **< 0.001** | **1.160 (1.151 to 1.168)** |
| Age groups |  |  | 10,454.620 | 7 | **< 0.001** |  |
| 40 to 59 years of age | 0.590 | 0.006 | 8443.551 | 1 | **< 0.001** | **1.803 (1.781 to 1.826)** |
| 60 to 64 years of age | 0.862 | 0.006 | 18,620.342 | 1 | **< 0.001** | **2.367 (2.338 to 2.397)** |
| 65 to 69 years of age | 1.092 | 0.006 | 28,440.467 | 1 | **< 0.001** | **2.979 (2.941 to 3.017)** |
| 70 to 74 years of age | 1.302 | 0.007 | 35,955.185 | 1 | **< 0.001** | **3.676 (3.627 to 3.726)** |
| 75 to 79 years of age | 1.528 | 0.007 | 43,640.087 | 1 | **< 0.001** | **4.609 (4.544 to 4.676)** |
| 80 to 84 years of age | 1.743 | 0.008 | 43,926.488 | 1 | **< 0.001** | **5.717 (5.627 to 5.810)** |
| 85 to 90 years of age | 2.091 | 0.009 | 49,839.238 | 1 | **< 0.001** | **8.093 (7.946 to 8.243)** |
| Race |  |  |  |  |  |  |
| White people |  |  | 6027.589 | 4 | **< 0.001** |  |
| Black people | 0.439 | 0.009 | 2332.266 | 1 | **< 0.001** | **1.552 (1.524 to 1.580)** |
| Asian individuals | 0.004 | 0.019 | 0.036 | 1 | 0.849 | 1.004 (0.967 to 1.042) |
| Mixed individuals^*^ | 0.271 | 0.004 | 4550.881 | 1 | **< 0.001** | **1.312 (1.301 to 1.322)** |
| Indigenous peoples | 0.417 | 0.044 | 89.661 | 1 | **< 0.001** | **1.517 (1.392 to 1.654)** |
| Place of residence (Urban) | -0.126 | 0.009 | 208.705 | 1 | **< 0.001** | **0.882 (0.867 to 0.897)** |
| Nosocomial infection | 0.202 | 0.013 | 228.705 | 1 | **< 0.001** | **1.224 (1.193 to 1.257)** |
| Clinical signs and symptoms |  |  |  |  |  |  |
| Cough | -0.163 | 0.005 | 1063.454 | 1 | **< 0.001** | **0.849 (0.841 to 0.858)** |
| Sore throat | -0.060 | 0.005 | 127.777 | 1 | **< 0.001** | **0.942 (0.933 to 0.952)** |
| Dyspnea | 0.218 | 0.005 | 1293.254 | 1 | **< 0.001** | **1.244 (1.229 to 1.258)** |
| Respiratory discomfort | 0.269 | 0.005 | 2540.991 | 1 | **< 0.001** | **1.309 (1.295 to 1.322)** |
| Peripheral oxygen saturation < 95% | 0.155 | 0.006 | 721.181 | 1 | **< 0.001** | **1.168 (1.155 to 1.181)** |
| Diarrhea | -0.099 | 0.006 | 265.298 | 1 | **< 0.001** | **0.906 (0.895 to 0.916)** |
| Vomiting | -0.019 | 0.008 | 6.192 | 1 | **0.013** | **0.981 (0.967 to 0.996)** |
| Fatigue | -0.135 | 0.005 | 844.445 | 1 | **< 0.001** | **0.874 (0.866 to 0.882)** |
| Comorbidities |  |  |  |  |  |  |
| Heart disease (cardiopathy) | -0.023 | 0.004 | 31.114 | 1 | **< 0.001** | **0.977 (0.970 to 0.985)** |
| Hematological disorder | 0.171 | 0.023 | 58.014 | 1 | **< 0.001** | **1.187 (1.136 to 1.240)** |
| Down syndrome | 0.246 | 0.041 | 36.314 | 1 | **< 0.001** | **1.279 (1.181 to 1.386)** |
| Hepatic disorder | 0.573 | 0.020 | 815.934 | 1 | **< 0.001** | **1.774 (1.705 to 1.845)** |
| Asthma | -0.245 | 0.013 | 349.362 | 1 | **< 0.001** | **0.783 (0.763 to 0.803)** |
| Diabetes mellitus | 0.142 | 0.004 | 1045.708 | 1 | **< 0.001** | **1.153 (1.143 to 1.163)** |
| Chronic lung disease | 0.189 | 0.009 | 403.130 | 1 | **< 0.001** | **1.208 (1.186 to 1.230)** |
| Immunosuppressive disease | 0.556 | 0.012 | 2159.842 | 1 | **< 0.001** | **1.744 (1.704 to 1.786)** |
| kidney disease | 0.502 | 0.010 | 2775.613 | 1 | **< 0.001** | **1.653 (1.623 to 1.684)** |
| Obesity | 0.178 | 0.007 | 603.553 | 1 | **< 0.001** | **1.195 (1.178 to 1.212)** |
| Received antiviral medication for the Flu | -0.246 | 0.008 | 991.086 | 1 | **< 0.001** | **0.782 (0.770 to 0.794)** |
| Needed intensive care unit | 0.721 | 0.005 | 23,866.288 | 1 | **< 0.001** | **2.057 (2.039 to 2.076)** |
| Needed ventilatory support |  |  |  |  |  |  |
| Invasive | 2.559 | 0.008 | 111,838.005 | 1 | **< 0.001** | **12.926 (12.734 to 13.121)** |
| Noninvasive | 0.620 | 0.006 | 11,185.562 | 1 | **< 0.001** | **1.859 (1.838 to 1.880)** |
| None |  |  | 138,981.088 | 2 | **< 0.001** |  |
| Period of time between ... |  |  |  |  |  |  |
| … date of admission and date of onset of symptoms | -0.019 | < 0.001 | 4606.670 | 1 | **< 0.001** | **0.980 (0.981 to 0.982)** |
| … date of outcome and date of admission | 0.012 | < 0.001 | 1636.187 | 1 | **< 0.001** | **1.013 (1.012 to 1.014)** |
| … date of discharge and date of admission to the intensive care unit | 0.004 | < 0.001 | 165.850 | 1 | **< 0.001** | **1.004 (1.003 to 1.005)** |
| Constant | -2.607 | 0.012 | 44,312.896 | 1 | **< 0.001** | **0.074** |

*: Mixed refers to individuals with a multiracial background (*Pardos*). β: the average increase in the outcome per unit increase of the predictor; DF: degrees of freedom; SE: standard error.

The association between markers is described using the Odds Ratio (OR) and the 95% confidence interval (95% CI). Multivariable analysis was performed using the Binary Logistic Regression Model with the Backward Stepwise method. Markers with a p-value ≤ 0.05 in the bivariate analysis were included in the regression model. The dependent variable was mortality.

Data for clinical signs and symptoms (others) and patient characteristics with a p-value > 0.05 were excluded from the analysis. A p-value of. 0.05 was considered statistically significant. Statistically significant data are marked in bold in the table for ease of interpretation.

The epidemiological analysis was based on data from Open-Data-SUS (https://opendatasus.saude.gov.br/), covering a 4-year period of the COVID-19 pandemic in Brazil (from February 22, 2020, to May 24, 2024).

^a^, Markers included in step 1 of the multivariable analysis: Parkinson’s disease diagnosis, COVID-19 vaccination status, sex, age group, race, place of residence, nosocomial infection, clinical signs and symptoms (fever, cough, sore throat, dyspnea, respiratory discomfort, peripheral oxygen saturation < 95%, diarrhea, vomiting, and fatigue), comorbidities [heart disease (cardiopathy), hematological disorder, Down syndrome, hepatic disorder, asthma, diabetes mellitus, chronic lung disease, immunosuppressive disease, kidney disease, and obesity], antiviral medication for the flu-like symptoms, need for intensive care unit admission, use of ventilatory support, time intervals between admission and symptom onset, outcome and symptom onset, outcome and admission, and discharge and intensive care unit admission.


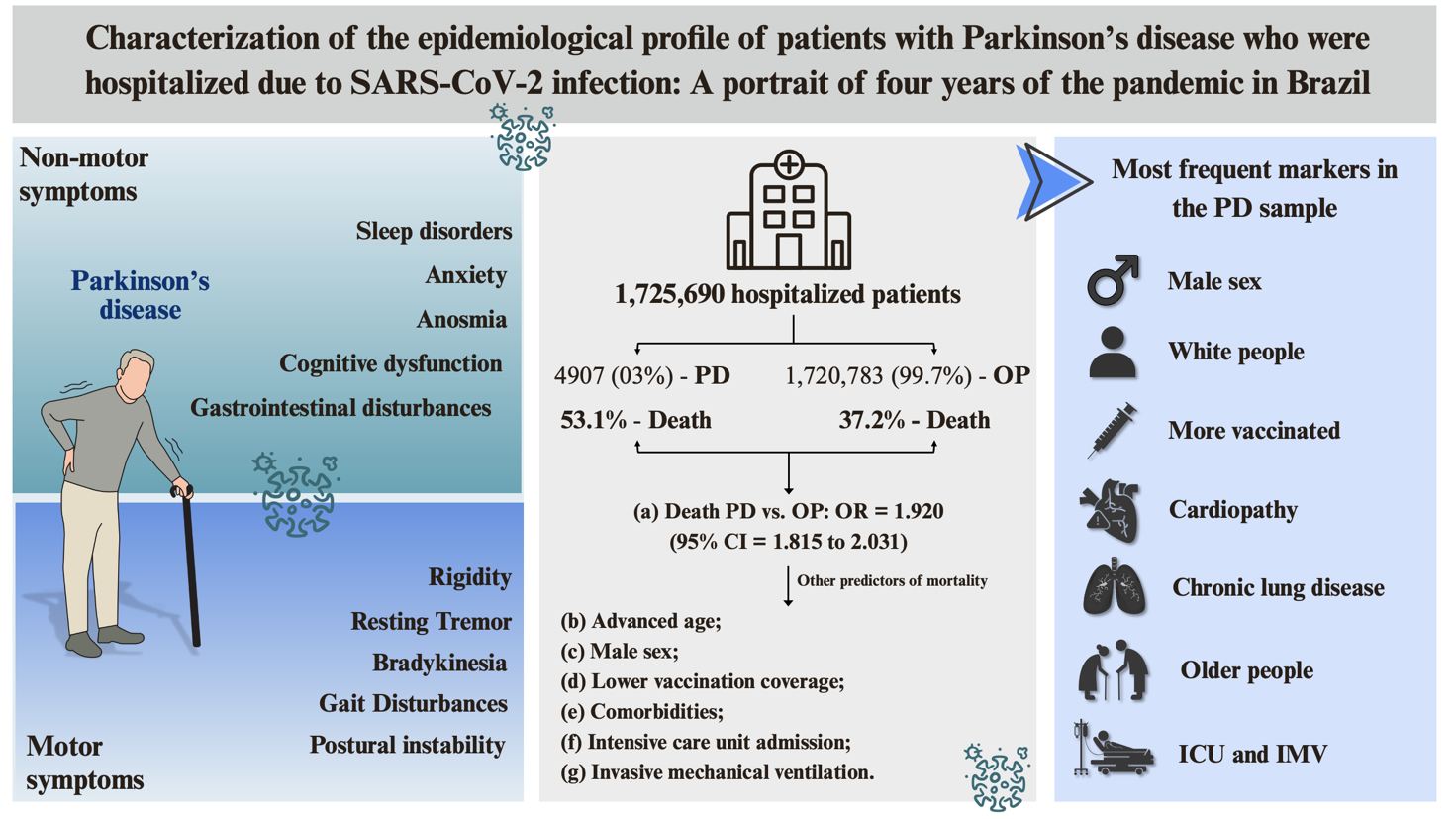


**Graph Abstract (Supplementary Figure 1).** Overview of the main findings of the study. 95% CI: 95% confidence interval; ICU: intensive care unit; IMV: invasive mechanical ventilation; OP: other patients; OR: odds ratio, PD: Parkinson’s disease; SARS-CoV-2: severe acute respiratory syndrome coronavirus 2. Data are presented as absolute frequency (N) and relative frequency (percentage, %).

The epidemiological analysis was based on data from Open-Data-SUS (https://opendatasus.saude.gov.br/), covering four years of the coronavirus disease (COVID)-19 pandemic in Brazil (from February 22, 2020, to May 24, 2024).
